# The prognostic and predictive value of AFP in immune checkpoint inhibitor-treated hepatocellular carcinoma: a systematic review and meta-analysis

**DOI:** 10.3389/fimmu.2025.1695861

**Published:** 2025-11-04

**Authors:** Bao-Wen Tian, Lun-Jie Yan, Wei-Chao Liang

**Affiliations:** ^1^ Department of General Surgery, The Sixth Affiliated Hospital, South China University of Technology, Foshan, Guangdong, China; ^2^ Department of General Surgery, Qilu Hospital of Shandong University, Jinan, China

**Keywords:** hepatocellular carcinoma, immune checkpoint inhibitor, alpha-fetoprotein, prognosis, meta-analysis

## Abstract

**Introduction:**

Alpha-fetoprotein (AFP) is a universally recognized tumor marker in hepatocellular carcinoma (HCC). Its utility in assessing the response to immune checkpoint inhibitors (ICIs) remains controversial. This study aims to investigate the predictive value of AFP in ICIs-treated HCC patients.

**Method:**

A systematic search strategy was deployed across the PubMed, Embase, Cochrane Library and Web of Science databases. Hazard ratios (HR) or odds ratios (OR) and the corresponding 95% confidence intervals (CIs) were used to assess the pooled risk.

**Result:**

The study encompassed a total of 131 studies. Overall survival (OS) (HR = 1.60, 95%CI=1.47-1.74), progression-free survival (PFS) (HR = 1.35, 95%CI=1.27-1.42), and disease control rate (DCR) (OR = 0.50, 95%CI=0.29-0.84) were poorer in ICIs-treated patients with high AFP levels than those with low AFP levels. However, AFP levels were not associated with the objective response rate (ORR) (OR = 0.96, 95%CI=0.74-1.24). In addition, patients who achieved an AFP response had favorable OS (HR = 0.41, 95%CI=0.33-0.52), PFS (HR = 0.38, 95%CI=0.30-0.47), ORR (OR = 5.39, 95%CI=3.96-7.32) and DCR (OR = 5.48, 95%CI=3.71-8.11). Subgroup analyses revealed that AFP>400ng/ml and AFP decline greater than 20% were the most used and efficient cut-off values for high AFP level and AFP response, respectively.

**Conclusion:**

High AFP levels are associated with worse outcomes in ICIs-treated HCC. The assessment of AFP response demonstrated promising predictive value for both prognosis and therapeutic response to ICIs. Accurately defining early AFP response remains an area that requires further investigation.

**Systematic Review Registration:**

https://www.crd.york.ac.uk/PROSPERO/, identifier CRD-42024606729.

## 1 Introduction

Primary liver cancer is the sixth most diagnosed tumor and the third leading cause of cancer-related deaths worldwide, with hepatocellular carcinoma (HCC) accounting for 75-85% of primary liver cancers (1). For the past 10 years, we have witnessed an evolution of systemic therapies for hepatocellular carcinoma. In particular, immune checkpoint inhibitors (ICIs) and other immunotherapies are revolutionizing cancer management (2–4). Numerous clinical studies (such as IMbravel150, ORIENT-32, CARES-310 and RATIONALE-301) have demonstrated that ICIs can improve the prognosis of HCC patients, leading to their widespread recommendation as a first-line therapy by prevailing guidelines (5–10). In advanced HCC, the combination of the anti-vascular endothelial growth factors antibody bevacizumab and the anti-programmed death ligand-1 (PD-L1) antibody atezolizumab has established a new first-line benchmark for reaching a median OS duration of 19 months, thus representing a breakthrough in the management of HCC (5). Despite systemic therapy especially ICIs treatment has started delivering unprecedented promising hope for HCC management, unfortunately, approximately 40% of HCC patients fail to achieve disease control due to primary resistance (11, 12). Therefore, predicting the treatment response and survival benefit at an early stage is becoming increasingly important for ICIs treatment of HCC.

Alpha-fetoprotein (AFP), the earliest discovered and most widely used serological marker for HCC diagnosis, plays a crucial role not only in diagnosing but also in evaluating the prognosis of HCC (13). AFP response is an established biomarker in HCC (14). The observation of a declining trend in serum AFP levels within the initial 4 to 8 weeks of treatment has been demonstrated to serve as a surrogate marker for improved overall survival (OS) among patients receiving chemotherapy and targeted therapies (15, 16). As for immunotherapy, high AFP levels have also been shown prognostic of survival outcomes (17–19). Moreover, recent studies demonstrate that early AFP response in the course of ICIs treatment may give clinicians an early hint of response or lack of response to immunotherapy in a proportion of HCC patients (20–22).

Nevertheless, the predictive value of AFP in immunotherapy has not been comprehensively evaluated. There is no universally accepted gold standard for establishing definitive criteria for high AFP levels or for determining optimal monitoring time points concerning dynamic changes in AFP. The assessment indicators most frequently employed in the evaluation of elevated AFP levels range from 100 to 400 ng/mL. With regard to the assessment of AFP response, temporal parameters vary across studies: some employ a 4-week time point, others use a 3-month interval, and in certain cases, no clearly defined time point is specified (23–25). Thus, we conducted this meta-analysis to elucidate the predictive significance of baseline AFP levels and AFP responses in ICIs-treated HCC, which may help determine the prognosis and formulate an effective treatment strategy.

## 2 Methods

This systematic review with meta-analysis was reported in accordance with the Preferred Reporting Items for Systematic Reviews and Meta-Analyses (PRISMA) statement guideline (26). The selection criteria were established based on the PICOS (population, intervention, comparison, outcome, and study design) framework. The systematic review was prospectively registered at PROSPERO as CRD-42024606729.

### 2.1 Data sources and search methods

We systemically searched four databases, namely PubMed, Embase, Cochrane Library, and Web of Science, from the time of their inception until October 31, 2024. We searched by key subject terms, including hepatocellular carcinoma, liver cancer, alpha-fetoprotein, immune checkpoint inhibitors, as well as specific ICIs such as nivolumab, pembrolizumab, atezolizumab, ipilimumab, etc. Moreover, a manual screening of reference lists of included studies was conducted to identify additional eligible publications. The PICOS model and detailed search strategies are provided in **Supplementary Retrieval Methods**.

### 2.2 Inclusion and exclusion criteria

The eligibility of all citations was assessed by two researchers (TBW and YLJ) independently. Divergences were resolved by discussion with another researcher (LT). To be qualified for inclusion, eligible research studies should meet the following inclusion criteria: (1) enrolled patients diagnosed with HCC, and received relevant ICIs, with or without additional therapies; (2) provided data about AFP (including AFP levels or AFP response) related to patient prognosis; (3) reported indicators related to treatment results, including overall survival (OS), progression-free survival (PFS), objective response rate (ORR) or disease control rate (DCR); and (4) reported hazard ratios (HR)/odds ratio (OR) with 95% confidence intervals (CI) which can be directly obtained.

Exclusion criteria were as follows: (1) the article was a case report, letter, animal trial, review, or conference abstract, etc.; (2) the study only reported survival curve and P value without HR and 95%CIs; (3) the study only enrolled a subset of patients received ICIs treatment but reported the prognostic data results from all participants; (4) for repeated publications and studies that include overlapping populations, only the latest and most comprehensive studies were included (but for studies with different indicators or different subgroups we included them for subgroup analysis); and (5) studies were not published in English.

### 2.3 Data extraction and quality assessment

Two investigators (TBW and YLJ) independently extracted data pertaining to the following items: first author, publication year, country/region, enrollment period, number of participants, gender ratio, mean/median age, intervention measures, combination therapy, previous therapy, subsequent therapy, data collection, study type, cutoff of AFP levels, definition of AFP levels, HR/OR and 95%CI. Univariate analysis and multivariate analysis data were extracted respectively. We used the Quality in Prognosis Studies (QUIPS) tool to evaluate the quality of the included studies (27). The QUIPS tool consists of six bias domains: study participation, study attrition, prognostic factor measurement, outcomes measurement, study confounding and statistical analysis and reporting. For each domain, the QUIPS tool employs a three-level classification system to assess the risk of bias, categorizing it as low, moderate or high. Disputes were resolved by discussion until a consensus was reached.

### 2.4 Statistical analysis

Statistical analyses were performed using Stata 17.0 (Stata Corp, College Station, Texas) statistical software. The random-effect model (DerSimonian–Laird method) was used for pooled analysis. A P value <0.05 was considered statistically significant. For studies providing both univariate and multivariate data, multivariate data were preferred for the pooled analysis in our study. Univariate data were considered if multivariate data were not performed. OS and PFS were used to evaluate the prognosis of HCC patients treated with ICIs. OS and PFS were estimated using HR and its corresponding 95%CI (HR >1 indicated a worse OS or PFS observed in the patients treated with ICIs). The inverse variance approach was used to construct study weights. Sensitivity analyses were performed to determine the stability of the pooled results and assess the robustness of the pooled effect. If the removal of one study outcome in the sensitivity analysis resulted in a significant bias of the pooled HR and 95%CI, the very outcome should be excluded.

We used Cochrane’s Q and the inconsistency index (I2) statistic to assess the statistical heterogeneity of the studies. Either I2 greater than 50% or P <0.10 was considered substantial or significant heterogeneity. In order to ascertain the potential sources of heterogeneity and refine the effect sizes under subgroups, univariate random-effects meta-regression models were constructed and subgroup analyses were performed. We used Funnel plots and Egger’s regression asymmetry test to examine the potential publication bias (28). The good symmetry of the funnel plot indicated that there was no obvious publication bias. Egger’s test was used to assess the symmetry of the funnel plots. We identified significant publication bias through funnel plots, and finally obtained the adjusted pooled HR and 95% CI using the trim-and-fill method to reduce publication bias.

## 3 Results

### 3.1 Literature search and baseline characteristics

**Figure 1:**
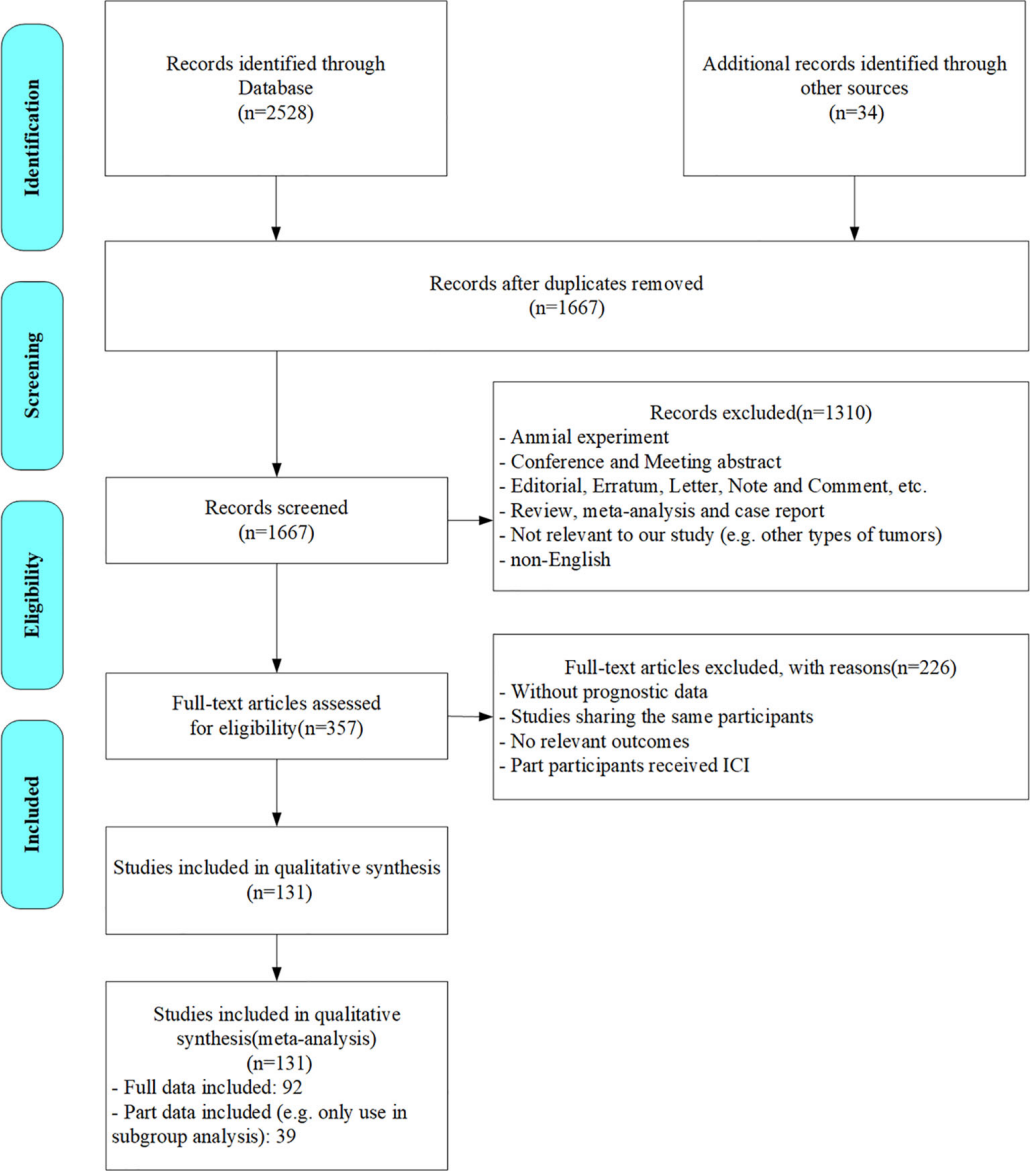
Flow diagram of study selection process.

According to the search strategy, 2562 citations were initially identified. We screened out 895 duplicates and further perused the titles and abstracts of the rest. After the preliminary exclusion of the citations with the inclusion criteria, 357 citations were left for further full-text review. Upon meticulous assessment, 226 studies were excluded for the following reasons: lacking specific data (e.g. prognostic data, AFP levels/AFP response data or HR/OR data), cohorts fully or partially overlapped with other studies (unless the study provided data under different subgroups or on different prognostic indicators), outcomes unrelated to the research focus, or only part of the participants received ICIs therapy. Ultimately, 131 studies were included in our analyses. The flowchart of the literature search process is presented in **Figure 1**.

The characteristics of the 131 studies are summarized in **Table 1** and **Supplementary Table 1**. Among these, 128 were cohort studies (122 retrospective and 6 prospective), and 3 were randomized controlled studies. The standard to define high AFP levels varied across studies (i.e. cut-point settings of 1000, 500, 400, 200, 100, 20ng/ml). Studies using cut-off values close but not exactly matching the aforementioned thresholds were integrated into the group using similar threshold (e.g. >101.4ng/ml was consolidated into >100ng/ml) for subgroup analysis. In most studies, AFP response was defined as a reduction of more than 20% in AFP levels within three months of initiating treatment. For the risk of bias assessment of all included studies, no studies had more than two domains with high risk. Therefore, all studies were assessed as having an overall low to moderate risk. The detailed results of the risk of bias assessment of the included studies are provided in **Supplementary Table S2**.

**Table 1:** The characteristics of included studies.

First author	Year	Region/ Country	Enrollment period	Patient No.	M/F	Age (mean/ median)	Data collection	Study type	AFP Response time	AFP Response cutoff	AFP Response exclude	AFP level
Y.-Y. Shao	2019	China (Taiwan)	2013-2018	43	35/8	55.0 (mean)	OS, PFS, ORR, DCR	retrospective	4 weeks	>20% decline	AFP<20 ng/ml	NA
S. Chen*	2020	China	2015.01- 2019.08	108	90/18	56 (median)	OS, PFS	retrospective	NA	NA	NA	≥200ng/ml
W. M. Choi	2020	Korea	2017.07- 2019.02	203	171/32	56.5 (mean)	ORR	retrospective	NA	NA	NA	≥400ng/ml
R. S. Finn	2020	Multicenter	2017.02- 2019.04	100	81/19	66.5 (median)	ORR	clinical trial	NA	NA	NA	≥200ng/ml
W. F. Hsu*	2020	China (Taiwan)	2017.05- 2019.12	87	79/8	63.4 (median)	OS, PFS, ORR	retrospective	3 months	≥20% decline	NA	≥400ng/ml
M. S. Lee	2020	Multicenter	2016.07- 2018.07	104	84/20	62 (median)	ORR	clinical trial	NA	NA	NA	≥400ng/ml
P. C. Lee*	2020	China (Taiwan)	2017.05- 2019.08	95	73/22	65.5 (median)	OS, ORR, DCR	retrospective	4 weeks	>10% decline	NA	>400ng/ml, <10ng/ml
S. Spahn	2020	Multicenter	2015.08- 2019.12	99	NA	69 (median)	OS, PFS	retrospective	NA	NA	NA	≥400ng/ml
P. S. Sung	2020	Korea	2016.10- 2019.11	33	25/8	57 (median)	OS	retrospective	NA	NA	NA	≥100ng/ml
G. Yuan*	2020	China	2019.01- 2020.07	63	58/5	48.7 (mean)	OS	retrospective	NA	NA	NA	≥400ng/ml
W. M. Choi	2021	Korea	2017.07- 2020.06	194	159/35	57.4 (mean)	OS	retrospective	NA	NA	NA	≥400ng/ml
W. F. Hsu*	2021	China (Taiwan)	2017.05- 2021.06	95	84/11	63.8 (median)	OS, PFS, ORR, DCR	retrospective	3 months	≥20%,15%, 10% decline	NA	≥400ng/ml
J. Mei*	2021	China	2018.06- 2019.12	70	56/14	49.5 (mean)	OS, PFS	retrospective	NA	NA	NA	≥400ng/ml
M. Morita	2021	Japan	2015.08- 2017.09	34	28/6	67.1 (median)	DCR	retrospective	NA	NA	NA	≥400ng/ml
K. Y. Y. Ng	2021	Singapore	2015.05- 2018.06	114	101/13	66 (median)	OS	retrospective	NA	NA	NA	≥400ng/ml
D. J. Pinato	2021	Multicenter	2017-2019	406	324/82	64 (median)	OS, PFS	retrospective	NA	NA	NA	≥400ng/ml
X. Sun*	2021	China	2018.01- 2019.12	235	204/31	51 (median)	OS, PFS, ORR	retrospective	6 weeks	>50% decline	AFP<25 ng/ml	>400ng/ml
W. Teng	2021	China (Taiwan)	2015-2019	90	68/22	61.4 (median)	OS, PFS, ORR, DCR	retrospective	4,12 weeks	≥10% decline, change with 50%	NA	≥400ng/ml
J. Chen*	2022	China	2018.07- 2019.11	101	82/19	50 (median)	PFS	retrospective	NA	NA	NA	>200ng/ml
S. C. Chen	2022	China (Taiwan)	2016.01- 2019.02	140	109/31	64.8 (mean)	ORR	retrospective	2,4 weeks	>10,50,66% decline	AFP<10 ng/ml	NA
J. Cheon*	2022	Korea	2020.05- 2020.11	121	101/20	61 (median)	OS, PFS	retrospective	NA	increase vs decrease	NA	≥400ng/ml
M. Chuma	2022	Japan	2020.10- 2021.06	94	73/21	73 (median)	ORR	retrospective	NA	NA	NA	≥38ng/ml
D. Dong	2022	China	2018.07- 2021.02	38	34/4	57 (median)	OS, PFS	retrospective	NA	NA	NA	>200ng/ml
Z. Guo	2022	China	2019.08- 2021.04	54	48/6	NA	OS, PFS, ORR	retrospective	NA	NA	NA	≥400ng/ml
Y. Hayakawa	2022	Japan	2020.10- 2021.04	52	42/10	73 (median)	ORR	retrospective	6 weeks	≥20% decline	NA	NA
J. T. Huang	2022	China	2019.01- 2021.12	64	55/9	57.9 (mean)	OS	retrospective	NA	NA	NA	>400ng/ml
R. Huang	2022	China	2019.02- 2020.09	110	100/10	54.5 (median)	OS, PFS	retrospective	NA	NA	NA	≥400ng/ml
S. Ju-1*	2022	China	2017.03- 2021.09	80	66/14	52 (median)	OS, PFS	retrospective	NA	NA	NA	≥200ng/ml
S. Ju-2	2022	China	2017.03- 2021.05	108	90/18	55,52 (median)	OS	retrospective	NA	NA	NA	≥200ng/ml
H. I. Kim	2022	Korea	2017.02- 2019.09	108	85/23	57 (median)	OS	retrospective	6-10, 14-18 weeks	>20% decline, >20% increase	NA	NA
H. S. Kim	2022	Korea	2012.06- 2018.03	261	219/42	59 (median)	OS, PFS, ORR	retrospective	NA	NA	NA	≥400ng/ml
S. W. Lee*	2022	China (Taiwan)	2019.04- 2021.07	33	26/7	66 (median)	ORR	retrospective	NA	>10% decline	NA	>400ng/ml
S. Lewis	2022	USA	2016.05- 2019.12	58	42/16	61.5 (mean)	OS	retrospective	NA	NA	NA	>101.8ng/ml
X. Li*	2022	China	2019.06- 2021.05	114	102/12	53 (median)	OS, PFS	retrospective	NA	>20% decline	AFP≤25 ng/ml	≥400ng/ml
H. Liu*	2022	China	2019.01- 2021.12	54	43/11	57.6 (mean)	PFS	retrospective	NA	NA	NA	≥400ng/ml
H. Matsumoto	2022	Japan	2020.10- 2022.02	32	19/13	77 (median)	PFS	retrospective	NA	NA	NA	≥400ng/ml
T.-R. Peng	2022	China (Taiwan)	2016.01- 2022.05	36	29/7	61.4 (mean)	ORR, DCR	retrospective	4 weeks	>10% decline	AFP≤10 ng/ml	NA
C. W. Su*	2022	China (Taiwan)	2016.01- 2019.10	29	20/9	61 (mean)	PFS	retrospective	NA	NA	NA	≥400ng/ml
X. Sun*	2022	China	2019.01- 2021.06	84	69/15	53 (median)	OS, PFS	retrospective	NA	NA	NA	≥400ng/ml
N. Tamaki	2022	Japan	2021.10- 2022.09	91	65/26	74 (median)	PFS, ORR, DCR	prospective	6 weeks	≥20%, 50%, 75% decline	AFP<10 ng/ml	NA
W. Teng	2022	China (Taiwan)	2020.09- 2022.01	89	75/14	61.3 (median)	OS, PFS, ORR	retrospective	6 weeks	≥75% decrease or ≤10% increase	NA	≥400ng/ml
Y. L. Wu*	2022	Multicenter	2019.01- 2022.04	296	245/51	66 (median)	OS, PFS	retrospective	NA	NA	NA	≥400ng/ml
Y. J. Xiang*	2022	China	2019-2020	103	85/18	NA	PFS	retrospective	NA	NA	NA	≥400ng/ml
J. Yao	2022	China	2018.04- 2021.07	136	115/21	58 (median)	OS, PFS, ORR	retrospective	NA	NA	NA	≥400IU/ml
R. You	2022	China	2019.08- 2021.03	101	89/12	56.8 (mean)	OS, PFS, ORR, DCR	prospective	NA	NA	NA	≥400ng/ml
Z. Zhang	2022	China	2018.01- 2020.08	101	84/17	55 (median)	OS, PFS	retrospective	NA	NA	NA	>200ng/ml
M. Zhao	2022	China	2018.01- 2020.12	160	129/31	58 (median)	OS, PFS	retrospective	NA	NA	NA	>400ng/ml
A. X. Zhu	2022	Multicenter	2018.03- 2019.01	150	122/28	62 (median)	OS, PFS	retrospective	6 weeks	≥75% decrease, ≤10% increase	AFP<20 ng/ml	NA
A. Akyildiz	2023	Turkey	2020.09- 2023.03	65	54/11	65 (median)	OS	retrospective	NA	NA	NA	≥200ng/ml
C. Campani-A	2023	France/ Italy	2020.04- 2022.02	38	30/8	61.42 (median)	OS, PFS, ORR	retrospective	3 weeks	≥20% decline	AFP<20 ng/ml	NA
C. Campani-B	2023	France/ Italy	2020.04- 2022.02	37	28/9	68.64 (median)	OS, PFS, ORR	retrospective	3 weeks	≥20% decline	AFP<20 ng/ml	NA
J. Cheon	2023	Korea	2020.05- 2021.08	169	139/30	61,62 (median)	OS, PFS	retrospective	NA	NA	NA	>400ng/ml
C. C. L. Cheung	2023	Singapore	2016.05- 2021.03	67	61/6	NA	OS	retrospective	NA	NA	NA	≥400ng/ml
H. C. Chiang	2023	China (Taiwan)	2016.11- 2021.02	88	70/18	60,65 (median)	OS	retrospective	NA	NA	NA	≥400ng/ml
T. Fukushima	2023	Japan	2020.10- 2021.10	150	120/30	72 (median)	OS, PFS	retrospective	NA	NA	NA	≥400ng/ml
C. Hong	2023	China	2018.08- 2020.10	215	189/26	NA	DCR	retrospective	NA	NA	NA	≥20ng/ml
W. F. Hsu	2023	China (Taiwan)	2017.05- 2022.03	110	94/16	64.5 (median)	OS, PFS, DCR	retrospective	3 months	>15% decline	NA	NA
G. Jia	2023	China	2016.01- 2022.03	117	106/11	58 (median)	OS, PFS	retrospective	NA	NA	NA	>400ng/ml
S. Kang	2023	USA	2015-2021	111	86/25	65 (median)	OS	retrospective	NA	NA	NA	≥400ng/ml
G. Li	2023	China	2018.03- 2020.10	39	37/2	56 (median)	OS	retrospective	1, 3 months	drop to normal, reduce by half	NA	≥400ng/ml
H. Li*	2023	China	2018.10- 2023.01	92	83/9	NA	OS, PFS	retrospective	NA	NA	NA	≥400ng/ml
J. Li*	2023	China	2019.04- 2020.12	110	94/16	56.2 (mean)	OS	retrospective	NA	NA	NA	>200ng/ml
Q. Li	2023	China	2018.02- 2019.02	98	66/32	52 (mean)	OS	retrospective	NA	NA	NA	≥20ng/ml
S. Li	2023	China	2018.10- 2022.04	102	90/12	NA	ORR	retrospective	NA	NA	NA	>20ng/ml
C. Liu	2023	China	2019.01- 2021.12	151	124/27	57.41 (mean)	OS, PFS	retrospective	NA	NA	NA	≥151.4ng/ml
T. Long*	2023	China	2019.03- 2021.08	81	71/10	495,49 (median)	OS, PFS	retrospective	NA	NA	NA	>400ng/ml
L. Lu*	2023	Multicenter	2018.03- 2019.01	264	216/48	59,67,65 (median)	OS, PFS	retrospective	NA	High-rising, Low-stable, Sharp-falling	NA	NA
M.-C. Luo*	2023	China	2020.08- 2022.11	77	68/9	55 (median)	PFS, ORR, DCR	retrospective	6±2 weeks	>50% decline	AFP<10 ng/ml	≥400ng/ml
H. Navadurong	2023	Thailand	2020.09- 2023.04	83	67/16	60.6 (mean)	OS	retrospective	NA	NA	NA	>500ng/ml
Y. Pan*	2023	China	2018.06- 2022.12	63	55/8	53.7 (mean)	OS, PFS	retrospective	NA	NA	NA	>20ng/ml
M. Persano*	2023	Multicenter	2018.05- 2022.05	823	657/166	NA	OS, ORR	retrospective	NA	NA	NA	≥400ng/ml
S. Qu	2023	China	2018.01- 2020.04	63	57/6	51 (median)	OS	prospective	NA	NA	NA	≥400ng/ml
R. Raj	2023	USA	2016-2022	96	78/18	67.1 (mean)	ORR	retrospective	NA	NA	NA	>400ng/ml
T. Sun	2023	China	2018.01- 2021.01	224	182/42	52.6 (mean)	OS, PFS	retrospective	NA	NA	NA	>400ng/ml
N. Tanabe	2023	Japan	2020.09- 2022.11	83	61/22	74 (median)	ORR, DCR	retrospective	3 weeks	>30% decline	AFP<20 ng/ml	≥400ng/ml
C. Tang	2023	China	2020.01- 2022.06	94	81/13	50,56 (median)	PFS	retrospective	NA	NA	NA	≥400ng/ml
J. Wang*	2023	China	2019.11- 2021.11	105	82/13	57.24 (mean)	OS, PFS	retrospective	NA	NA	NA	≥400ng/ml
Y. L. Wu	2023	Multicenter	2017-2019	578	464/114	65 (median)	OS, PFS, ORR	retrospective	NA	NA	NA	>400ng/ml
Y. Xiao*	2023	China	2020.10- 2022.04	88	75/13	53 (median)	OS, PFS	retrospective	NA	NA	NA	≥400ng/ml
H. Xin	2023	China	2019.01- 2021.10	137	123/14	50.82 (mean)	PFS	retrospective	NA	NA	NA	>400ng/ml
L. Xu*	2023	China	2018.01- 2020.12	85	76/9	53.25 (mean)	OS, PFS	retrospective	NA	NA	NA	≥200ng/ml
M. H. Xu	2023	China	2018.10- 2022.02	210	188/22	57 (median)	OS, PFS	retrospective	NA	NA	NA	>400ng/ml
X. Yang	2023	China	2019.01- 2022.04	46	39/7	49,53 (median)	OS, PFS	retrospective	NA	NA	NA	>400ng/ml
Y. Yano	2023	Japan	2020.11- 2022.09	139	107/32	73.1 (mean)	OS	retrospective	NA	NA	NA	>400ng/ml
Y. Yin	2023	China	2019.01- 2021.07	44	35/9	62.5,55 (median)	OS, PFS	retrospective	NA	NA	NA	≥400ng/ml
B. Yu	2023	China	2020.12- 2022.07	748	649/99	NA	OS, PFS	retrospective	NA	NA	NA	>400ng/ml
W. Zhang-1*	2023	China	2019.07- 2021.02	56	NA	NA	OS, PFS	clinical trial	NA	NA	NA	≥400ng/ml
W. Zhang-2*	2023	China	2018.11- 2021.12	135	111/24	58 (median)	OS, PFS	retrospective	NA	NA	NA	≥400ng/ml
Y. Zhang	2023	China	2019.01- 2023.01	84	75/9	52 (median)	PFS	retrospective	8 week	>20% decline	NA	≥400ng/ml
H. F. Zhu	2023	China	2019.03- 2021.06	149	125/24	56 (median)	OS, PFS	retrospective	NA	NA	NA	>400ng/ml
H. Cai*	2024	China	2019.09- 2022.09	30	28/2	55.5 (median)	PFS	retrospective	NA	NA	NA	≥400ng/ml
B. B. Chen	2024	China (Taiwan)	2015.08- 2022.02	143	124/19	59.8 (mean)	PFS	retrospective	NA	NA	NA	>400ng/ml
J. L. Chen	2024	China	2021-2023	124	112/12	55 (median)	OS	retrospective	NA	NA	NA	≥400ng/ml
Y. Chen*	2024	China	2020.11- 2022.06	56	49/7	NA	OS, PFS	retrospective	NA	NA	NA	≥400ng/ml
M. Chuma	2024	Japan	2020.10- 2022.08	134	108/26	71 (median)	OS, PFS	prospective	NA	NA	NA	>100ng/ml
F.-D. Copil	2024	France	2020.09- 2023.05	295	247/48	66 (median)	OS, PFS	retrospective	NA	NA	NA	≥400ng/ml
L. Diao	2024	China	2020-2022	121	99/22	60.3 (mean)	OS, PFS	retrospective	NA	NA	NA	≥400ng/ml
S. Fu	2024	China	2020.11- 2023.06	91	81/10	NA	OS, PFS	retrospective	NA	NA	NA	≥400ng/ml
Y. Guo*	2024	China	2018.05- 2022.01	98	86/12	57 (mean)	OS, PFS	retrospective	NA	NA	NA	≥400ng/ml
J. Han	2024	China	2021.04- 2023.12	155	137/18	56.58 (mean)	PFS	retrospective	NA	NA	NA	>400ng/ml
M. He	2024	China	2018.11- 2021.12	102	96/6	NA	OS, PFS	retrospective	NA	NA	NA	>400ng/ml
Z. Huang	2024	China	2019.05- 2022.10	123	109/14	NA	OS	retrospective	NA	NA	NA	>400ng/ml
M. Kai	2024	Japan	2020.11- 2022.08	222	176/46	73 (median)	OS, PFS	prospective	NA	NA	NA	≥400ng/ml
S. Kaneko	2024	Japan	2020.11- 2023.03	213	183/30	74 (median)	OS	retrospective	NA	NA	NA	≥100ng/ml
T. Kuzuya	2024	Japan	2023.03- 2024.05	40	33/7	75 (median)	PFS	retrospective	NA	NA	NA	≥100ng/ml
S.-W. Lee	2024	China (Taiwan)	2018.06- 2020.05	57	48/9	66.7 (mean)	OS, ORR, DCR	retrospective	NA	>10% decline	NA	>400ng/ml
J. Li	2024	China	2020.01- 2021.12	119	105/14	56 (median)	OS, PFS	retrospective	NA	>18% decline	NA	NA
R. Li*	2024	China	2019.01- 2021.12	162	137/25	NA	OS, PFS	retrospective	6 week	>20% decline	AFP<20 ng/ml	>400ng/ml
Y. Li	2024	China	2020.01- 2021.12	166	153/13	52,51 (median)	PFS	retrospective	NA	NA	NA	>400ng/ml
K.-Y. Lin*	2024	China	2019.11- 2022.08	74	60/14	52.6 (mean)	PFS	retrospective	NA	>80%,50%, 20% decline	AFP<20 ng/ml	≥1000ng/ml
J. Liu	2024	China	2019.06- 2022.12	120	102/18	NA	OS, PFS	retrospective	NA	NA	NA	>400ng/ml
Y. Lu	2024	China	2020.01- 2023.12	98	87/11	59.5 (median)	OS, PFS	retrospective	NA	NA	NA	≥400ng/ml
K. P. Ma*	2024	China	2019.03- 2022.04	102	89/13	57.64 (mean)	OS, PFS	retrospective	NA	>20% decline	NA	>400ng/ml
W. Ma	2024	China	2020.02- 2022.11	51	44/7	59 (mean)	OS, PFS	retrospective	NA	NA	NA	≥200ng/ml
Z. Mo	2024	China	2016.03- 2022.12	168	136/32	54.13 (mean)	OS	retrospective	NA	NA	NA	>400ng/ml
E. Moriyama	2024	Japan	2020.11- 2024.06	109	85/24	74 (median)	OS	retrospective	NA	NA	NA	≥400ng/ml
T. Nakabori*	2024	Japan	2020.11- 2022.09	29	27/2	75 (median)	OS, PFS	retrospective	NA	NA	NA	≥200ng/ml
M. Nakazawa	2024	USA	2017.01- 2023.12	36	21/15	65 (median)	PFS	retrospective	NA	NA	NA	≥400ng/ml
F. Rossari	2024	Multicenter	2020.05- 2022.04	885	705/180	72 (median)	OS, PFS	retrospective	NA	NA	NA	≥400ng/ml
I. Saeki	2024	Japan	2023.03- 2023.09	110	90/20	72 (median)	ORR, DCR	retrospective	4 weeks	≥10% decline	NA	≥400ng/ml
R. Sobirey	2024	USA	2017.01- 2023.01	37	33/4	69.1 (mean)	OS	retrospective	NA	NA	NA	≥500ng/ml
W. Sun	2024	China	2019.07- 2022.01	180	151/29	57.5 (median)	OS, PFS	retrospective	NA	NA	NA	≥400ng/ml
T. Tada	2024	Japan	2018.05- 2023.10	936	740/196	74 (median)	OS, PFS	retrospective	NA	NA	NA	≥100ng/ml
L. Wang*	2024	China	2019.06- 2021.10	93	86/7	54.5 (mean)	OS, PFS	retrospective	NA	NA	NA	≥400ng/ml
Y. Q. Wang	2024	China	2019.01- 2023.12	126	80/46	NA	OS, PFS	retrospective	NA	NA	NA	>1210ng/ml
Y. Xiao*	2024	China	2020.10- 2022.04	63	52/11	53 (median)	OS, PFS, ORR, DCR	retrospective	4, 8 weeks	>20%,75% decline	NA	≥400ng/ml
Y. Xin	2024	China	2020.01- 2021.12	45	37/8	55.8 (mean)	OS, PFS	retrospective	NA	NA	NA	≥400ng/ml
L. Xu	2024	China	2020.09- 2022.01	64	53/11	52.5 (median)	ORR	prospective	NA	NA	NA	≥400ng/ml
J. Yang*	2024	China	2019.01- 2022.06	61	57/4	53 (median)	PFS	retrospective	NA	increase	NA	≥400ng/ml
Y. Yao	2024	China	2017.01- 2022.07	117	92/25	NA	OS, PFS	retrospective	NA	NA	NA	≥400ng/ml
X. Zheng	2024	China	2018.01- 2023.02	194	185/9	52.83 (mean)	OS, PFS	retrospective	4 weeks	>20% decline	NA	>400ng/ml
M. Zuo	2024	China	2019.04- 2022.10	416	373/43	50.7 (mean)	OS, PFS	retrospective	NA	NA	NA	>400ng/ml

M/F, male/female; AFP, alpha-fetoprotein; OS, overall survival; PFS, progression-free survival; ORR, objective response rate; DCR, disease control rate; NA, not available.

*: Studies were only partially included (e.g. only used as partial subgroup analyses) due to partial duplication of populations with other studies that were included in the analysis.

### 3.2 Impact of baseline AFP levels on OS and PFS in ICI-treated HCC

**Figure 2:**
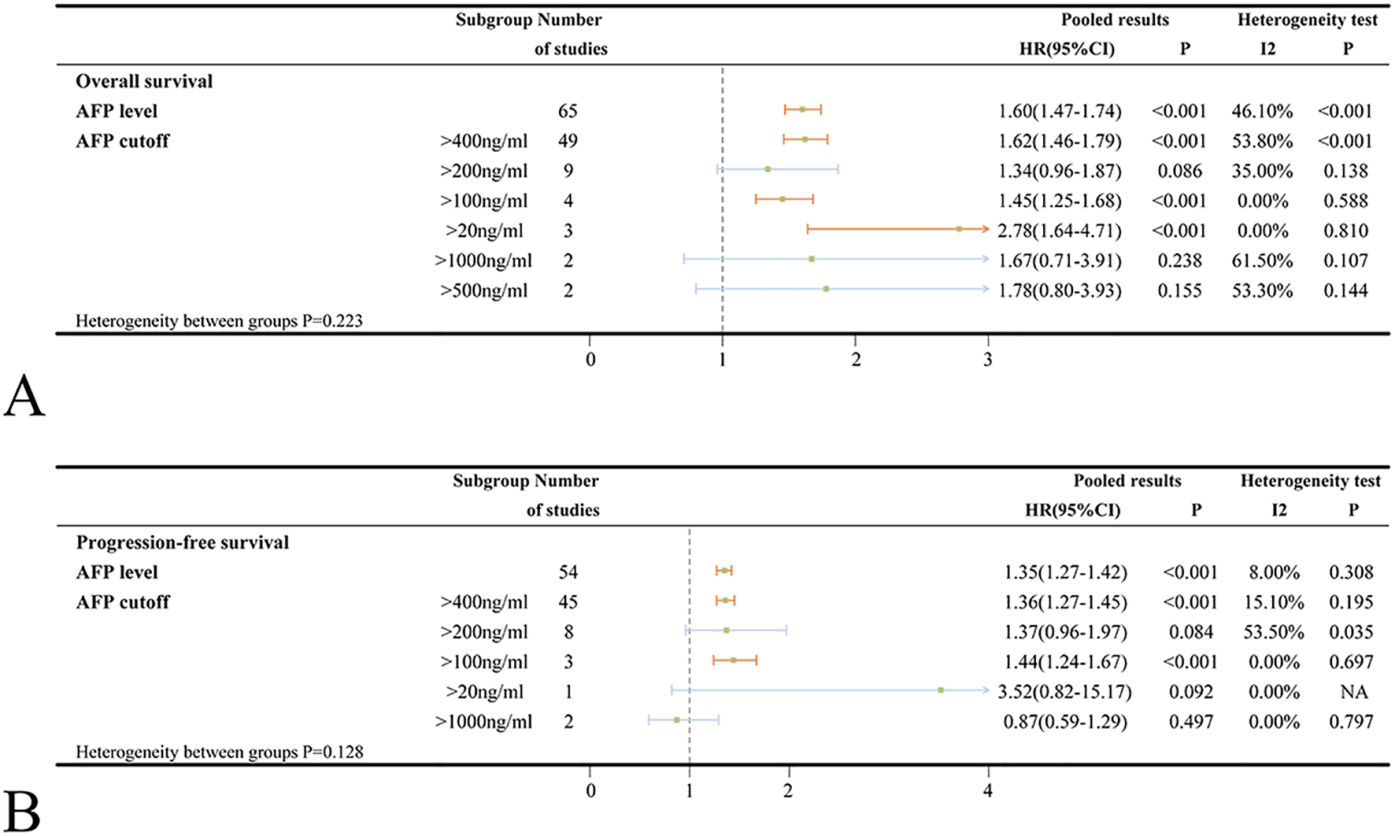
**(A)** Forest plots of OS in ICIs-treated HCC patients with high AFP level; **(B)** Forest plots of PFS in ICIs-treated HCC patients with high AFP level (HR>1 means the patients had worse OS or PFS). OS, overall survival; PFS, progress-free survival; ICI, immune checkpoint inhibitor; HCC, hepatocellular carcinoma; NA, not available.

Sixty-five studies investigated the association between baseline AFP levels and OS in ICIs-treated HCC. The pooled HR for the OS outcome in patients with high AFP levels was 1.60 (95%CI=1.47-1.74, *p* < 0.001) compared to those with low AFP levels. When stratified by AFP cut-off value, patients with high AFP levels at cut-off value of 400ng/ml (HR = 1.62, 95%CI=1.46-1.79, *p* < 0.001) had significantly poorer OS compared to those with low AFP levels (**Figure 2A**).

Significant heterogeneity was noted (*I* ^2^ = 46.1%, *p* < 0.001). To explore potential sources of the heterogeneity, several covariates were examined using meta-regression; however, there were no specific factors to blame (**Supplementary Table S3**). The sensitivity analysis also indicated that no individual study was accountable for the excess heterogeneity (**Supplementary Figure S1A**). Given the observed heterogeneity, subgroup analyses were performed by AFP cut-off value, country/region, patient number, age, medication, combination treatment, study type and effect size to assess the robustness of the conclusion (**Figure 2A**, **Supplementary Figure S2A**). Subgroup analyses stratified by country/region, patient number and patient age did not reveal any significant differences between subgroups (heterogeneity p-values: 0.857, 0.448 and 0.582; **Supplementary Figure S2A**). However, high AFP levels were found to be more strongly associated with worse OS in the Japanese study (HR = 1.85) compared to the studies conducted in China’s mainland (HR = 1.58) or Taiwan, China (HR = 1.53). Among different types of ICIs, the pooled HRs for the OS outcomes were 1.69, 1.68, 1.36 in patients treated with camrelizumab, atezolizumab and nivolumab, respectively, when comparing high AFP levels to low levels (**Supplementary Figure S2A**). Regarding combination treatment, high AFP levels did not significantly associate with poorer OS in patients receiving concurrent treatment with ICIs and hepatic arterial infusion chemotherapy (HAIC) (HR = 1.54, 95%CI=0.90-2.65, *p* = 0.115), ICIs combined with radiotherapy (HR = 1.44, 95%CI=0.85-2.42, *p* = 0.175) or ICIs combined with apatinib (HR = 1.88, 95%CI=0.69-5.10, *p* = 0.217). The association between high AFP levels and poorer OS had been validated across subgroups stratified by study design (retrospective studies: HR = 1.60, prospective studies: HR = 1.69) and regression type (univariate analysis: HR = 1.72, multivariate analysis: HR = 1.71) (**Supplementary Figure S2A**).

Data on PFS of ICIs for HCC were provided in 54 studies. Patients with high AFP levels exhibited significantly worse PFS compared to those with low AFP levels (HR = 1.35, 95%CI=1.27-1.42, *p* < 0.001) with no significant heterogeneity observed (*I* ^2^ = 8.0%, *p* = 0.308). Similarly, patients with high AFP levels at cut-off value of 400ng/ml had significantly poorer PFS (HR = 1.36, 95%CI=1.27-1.45, *p* < 0.001) compared to those with low AFP levels (**Figure 2B**).

When examining different medications, high AFP levels were found to be significantly related to worse PFS exclusively in patients treated with atezolizumab (HR = 1.39, 95%CI=1.26-1.53, *p* < 0.001). Conversely, no statistically significant associations were observed for camrelizumab (HR = 1.27, 95%CI=0.99-1.62, *p* = 0.058) or nivolumab (HR = 1.21, 95%CI=0.90-1.63, *p* = 0.204) (**Supplementary Figure S2B**). Furthermore, high AFP levels were not significantly associated with worse PFS in combination treatments involving ICIs plus TACE (HR = 1.23, 95%CI=0.94-1.62, *p* = 0.137) or ICIs plus apatinib (HR = 1.18, 95%CI=0.57-2.44, *p* = 0.649). However, high AFP levels were significantly associated with worse PFS in ICIs plus HAIC (HR = 1.81, 95%CI=1.27-2.57, *p* = 0.001) (**Supplementary Figure S2B**). No significant differences were observed between subgroups stratified by patient number (<100 or ≥100) and mean/median patient age (<60 or ≥60) (**Supplementary Figure S2B**). What’s more, the results from prospective studies were not statistically significant (HR = 1.10, 95%CI=0.84-1.44, *p* = 0.510). In contrast, retrospective studies, univariate and multivariate analyses yielded statistically significant results (**Supplementary Figure S2B**).

### 3.3 Impact of AFP response on OS and PFS in ICI-treated HCC

**Figure 3:**
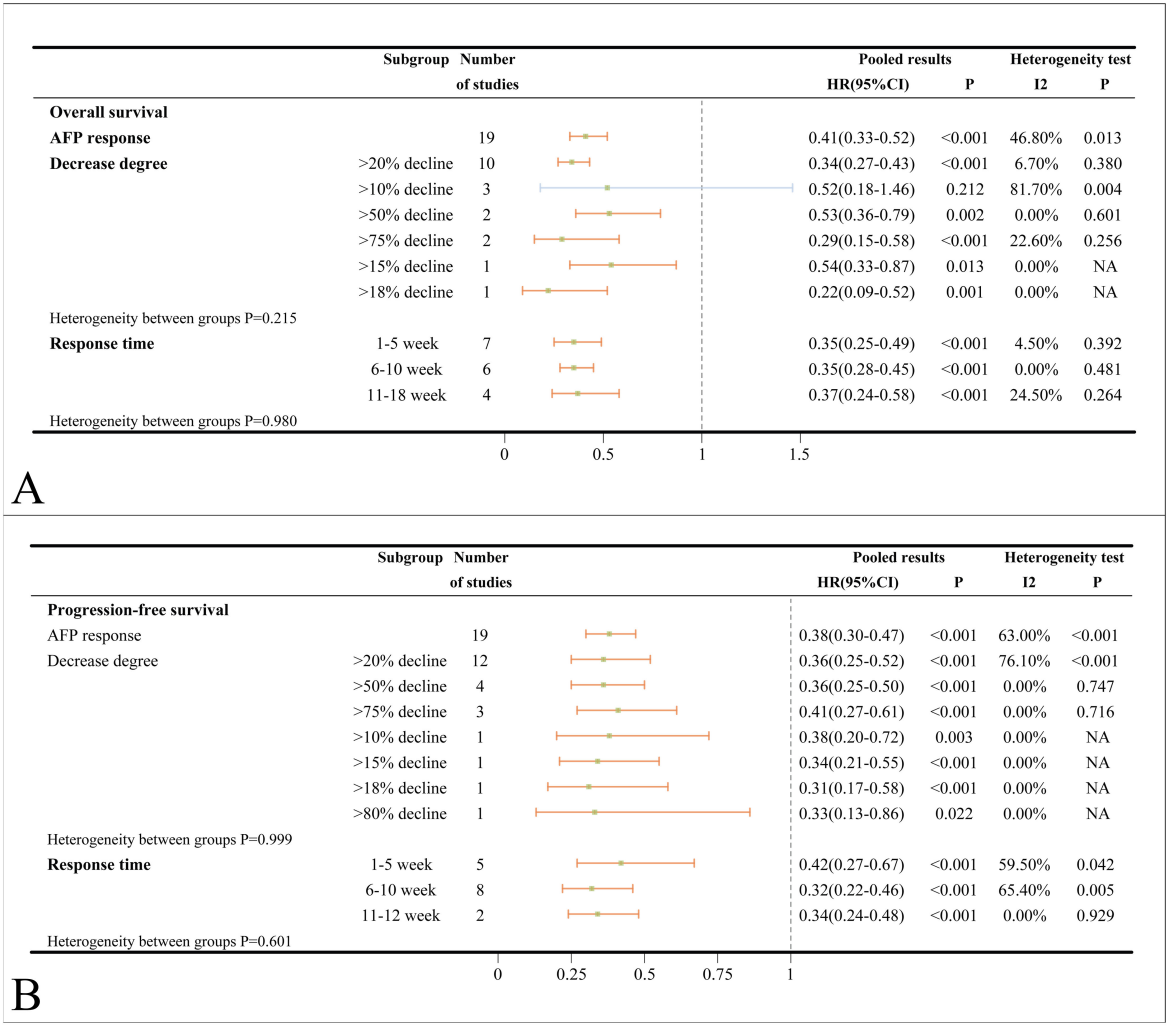
**(A)** Forest plots of OS in ICIs-treated HCC patients with AFP response; **(B)** Forest plots of PFS in ICIs-treated HCC patients with AFP response (HR>1 means the patients had worse OS or PFS). OS, overall survival; PFS, progress-free survival; ICI, immune checkpoint inhibitor; HCC, hepatocellular carcinoma; NA, not available.

A total of 19 studies investigated the association between AFP response and OS. When multiple criteria were used to define AFP response within a single study, an initial analysis prioritized a decrease in AFP greater than 20% as the primary criterion. The pooled HR for OS in ICIs-treated HCC patients who exhibited an AFP response was 0.41 (95%CI=0.33-0.52, *p* < 0.001), implying that patients with an AFP response derive greater benefit from ICIs than non-responders. Substantial heterogeneity was detected in the analysis (*I* ^2^ = 46.8%, *p* = 0.013). Subgroup analyses based on the degree of AFP decline revealed the strongest association for a decline exceeding 75% (HR = 0.29), followed by declines greater than 20% (HR = 0.34) and greater than 50% (HR = 0.53). An AFP decline of more than 10% after ICIs treatment did not indicate prolonged OS (HR = 0.52, 95%CI=0.18-1.46, *p* = 0.212) (**Figure 3A**). Consistent results were observed across subgroups stratified by time points (1–5 weeks, 6–10 weeks, and 11–18 weeks) for assessment (**Figure 3A**). Substantial heterogeneity was detected in the main analysis (I2 = 46.8%, p=0.013). However, sensitivity analysis confirmed the robustness of these findings, as no individual study was suspected of causing excess heterogeneity (**Supplementary Figure S5A**). In subgroup analyses based on different types of ICIs, there was no statistically significant correlation between AFP response and OS in patients treated with nivolumab (HR = 0.68, 95%CI=0.14-2.86, *p* = 0.562). Additionally, AFP response was not significantly associated with worse OS in the combination of ICIs and TACE (HR = 0.57, 95%CI=0.12-2.59, *p* = 0.463) (**Supplementary Figure S6A**). In subgroup analyses based on country/region, patient number and patient age, only the Korean subgroup (HR = 0.52, 95%CI=0.23-1.20, *p* = 0.125) did not reach statistical significance (**Supplementary Figure S6A**). The association between AFP response and improved OS was validated in both univariate analyses subgroup (HR = 0.48) and multivariate analyses subgroup (HR = 0.37).

The pooled HR for PFS of ICIs-treated HCC with AFP response was 0.38 (95%CI=0.30-0.47, *p* < 0.001) compared to those without. Substantial heterogeneity was detected in the analysis (*I* ^2^ = 63.0%, *p* < 0.001). Subgroup analysis by different degrees of AFP decline (>20%, >50% and >75%) and different time points for evaluation (1–5 weeks, 6–10 weeks, and 11–12 weeks) consistently supported the sentiment that AFP response can reliably predict improved PFS (**Figure 3B**). Furthermore, subgroup analyses stratified by country/region, patient number, patient age, medication types and treatment combinations had revealed that AFP response is associated with superior PFS (**Supplementary Figure S6B**). Consistent results were observed in retrospective studies (HR = 0.38), univariate analyses (HR = 0.39) and multivariate analyses (HR = 0.44), all of which were also statistically significant (**Supplementary Figure S6B**).

### 3.4 Impact of AFP value and AFP response on ORR and DCR

**Figure 4:**
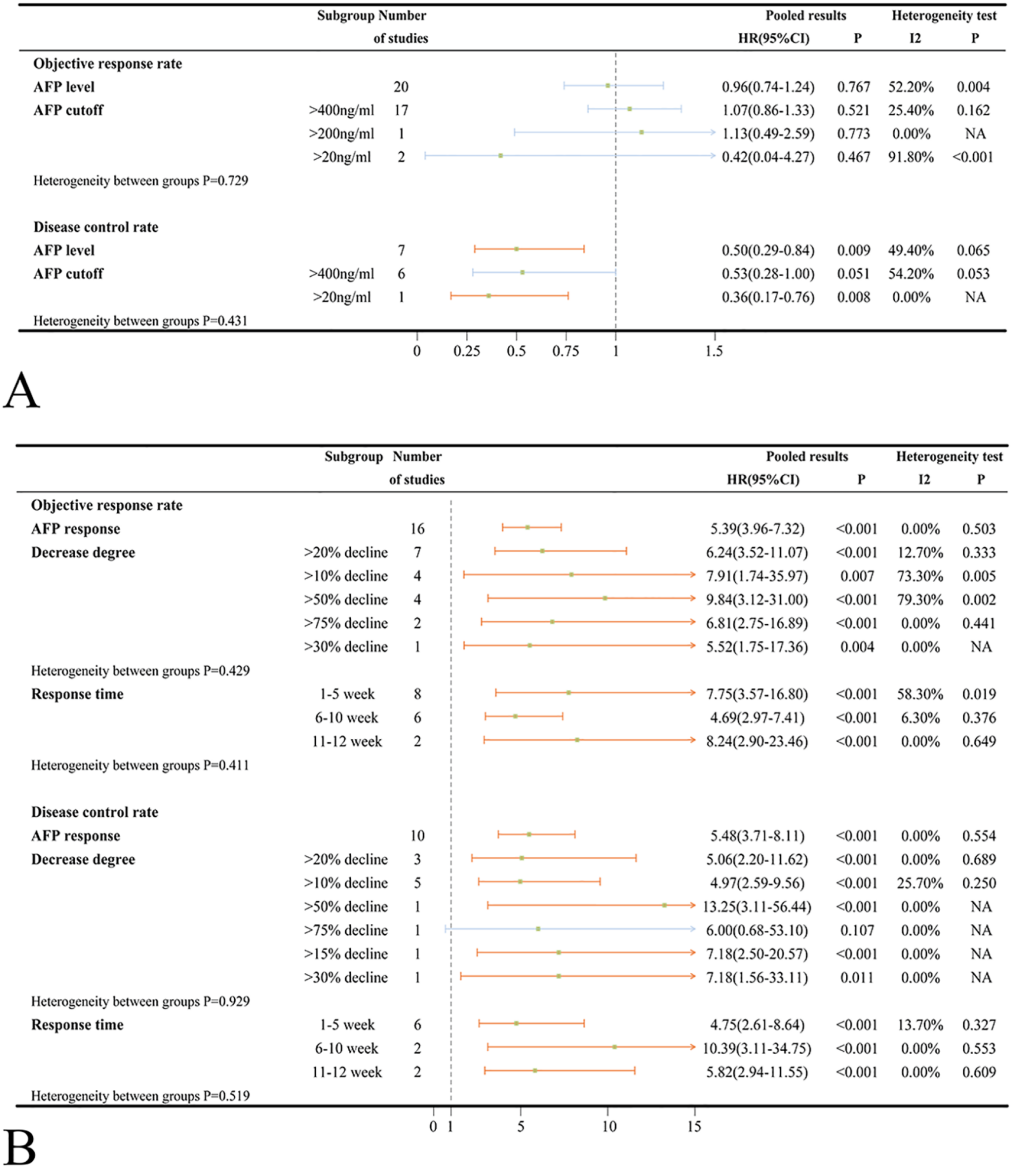
**(A)** Forest plots of ORR and DCR results in ICIs-treated HCC patients with high AFP level; **(B)** Forest plots of ORR and DCR results in ICIs-treated HCC patients with AFP response (OR>1 means the patients had well ORR or DCR). ORR, objective response rate; DCR, disease control rate; ICI, immune checkpoint inhibitor; HCC, hepatocellular carcinoma.

Various studies reported ORR and DCR based on the Response Evaluation Criteria in Solid Tumors (RECST v1.1) and modified RECST (mRECST) criteria. Data on ORR and DCR were primarily analyzed using the mRECST standard. High AFP levels did not correlate significantly with ORR (OR = 0.96, 95%CI=0.74-1.24, *p* = 0.767) but were associated with a worse DCR (OR = 0.50, 95%CI=0.29-0.84, *p* = 0.009) in ICIs-treated HCC patients (**Figure 4A**). When stratified by an AFP cut-off value of 400 ng/ml, high AFP levels were not associated with worse ORR (OR = 1.07 95%CI=0.86-1.33, *p* = 0.521) and DCR (OR = 0.53, 95%CI=0.28-1.00, *p* = 0.051) (**Figure 4A**). Subsequent subgroup analyses categorized according to country/region, patient number, patient age, medication, and study type revealed no significant between-group heterogeneity (**Supplementary Figure S9A**). Based on mRECIST, neither ORR (OR = 0.87, 95%CI=0.57-1.35, *p* = 0.544) nor DCR (OR = 0.64, 95%CI=0.17-2.42, *p* = 0.513) reached statistical significance. However, under RECIST standard, DCR was statistically significant (OR = 0.45, 95%CI=0.28-0.72, *p* = 0.001), while ORR was not (OR = 1.16, 95%CI=0.93-1.43, *p* = 0.188) (**Supplementary Figure S9A**). It was important to note that substantial heterogeneity was detected in both ORR (*I* ^2^ = 65.1%, *p* = 0.001) and DCR (*I* ^2^ = 75.0%, *p* = 0.018) subgroup when assessed by mRECIST standard.

HCC patients treated with ICIs who exhibited an AFP response had significantly higher ORR (OR = 5.39, 95%CI=3.96-7.32, *p* < 0.001) and DCR (OR = 5.48, 95%CI=3.71-8.11, *p* < 0.001) compared to those without an AFP response (**Figure 4B**). Different evaluation time points (1–5 weeks, 6–10 weeks and, 11–12 weeks) and criteria (RECIST and mRECIST) landed credence to the notion that AFP response can reliably predict improved ORR and DCR (**Figure 4B**, **Supplementary Figure S9B**). Similar to the findings in patients with high AFP levels, there was no statistically significant heterogeneity between subgroups based on country/region, patient number, patient age, medication, and study type (**Supplementary Figure S9B**).

### 3.5 Publication bias

Funnel plot and Egger’s linear regression test were employed to detect the potential publication bias. Funnel plots and Egger’s test indicated potential publication bias in the OS result based on AFP levels. In contrast, the funnel plots for other main results were approximately symmetrical (**Supplementary Figures S9**, **S10**). The results of Egger’s test, along with the adjusted pooled HR and 95% CI obtained using the trim-and-fill method, are summarized in **Supplementary Tables S4** and **S5**. The metatrim results did not significantly alter the main conclusions.

## 4 Discussion

The main categories of indicators used to predict the efficacy of ICIs include blood or cellular biomarkers, tumor-related biomarkers, imaging and physical markers, HCC etiology, intestinal flora, and immune-related adverse events (29, 30). AFP is the most commonly utilized serological indicator in the clinical management of HCC, favored for its broad applicability and relatively low cost. Many studies have found that high AFP levels are associated with poorer OS and PFS in ICIs-treated HCC patients (31, 32). Furthermore, an increasing number of studies are exploring the development of more comprehensive prognostic scores based on baseline AFP levels, such as the CRAFITY, TAE and α-FAtE scores etc. (33–37) Further large-scale clinical studies are required to validate the validity and reliability of these indicators.

The findings from our previous meta-analysis suggested that a 20% reduction in AFP within eight weeks following systemic therapy could serve as a reasonably precise criterion for an early AFP response (38). However, only eight studies related to ICIs treatment were included in that analysis. Given the increasing prevalence of ICIs in the treatment of HCC, there has been a corresponding rise in studies evaluating the prognostic value of AFP response in ICIs-treated patients (23, 25). It is necessary to further validate the pre-conclusions in the context of ICIs treatment.

Our primary objective was to evaluate the association between high baseline AFP levels and patient prognosis. No significant differences were observed in short-term efficacy metrics between HCC patients treated with ICIs who had high versus low AFP levels. Nevertheless, regarding long-term prognosis, patients with high AFP levels exhibited comparatively reduced OS and PFS. According to subgroup analysis in our study, it can be reasonably proposed that cut-off values of 400ng/ml may be more appropriate for use in the prognostic scoring system based on baseline AFP.

Following further validation, the AFP response may serve as a reliable predictor of both short-term and long-term efficacy of ICIs treatment. HCC patients treated with ICIs who exhibited an AFP response demonstrated higher proportion of ORR and DCR as determined by imaging assessment. Additionally, these patients showed prolonged PFS and OS. Despite variations in the timing of AFP response evaluation across different studies, our findings indicate that there is no notable discrepancy in the impact of different time points within 3 months on the assessment of AFP response. A 20% decline is widely accepted as a criterion for determining the extent of decline. Though a few studies have suggested that patients with a 10% deduction or even smaller declines may have better prognoses. Further studies are required to confirm this conclusion (17, 39, 40). Given the dynamic nature of AFP levels following ICI treatment, adopting a dynamic monitoring approach will facilitate a more comprehensive understanding of the AFP response (41, 42). In consideration of the existing research data, it is currently not feasible to formulate a universally accepted definition of early AFP response. Therefore, we recommend that future studies conduct a more rigorous comparison of the differences between various criteria for evaluating AFP response.

Our meta-analysis provides a comprehensive summary of the current data on the baseline AFP levels and AFP response in ICIs-treated HCC patients. Nevertheless, it is important to acknowledge that this study has several inherent limitations. First, the majority of the included studies were retrospective, which may introduce potential biases and inaccuracies in the original data. Second, most patients received additional treatments concurrently with ICIs treatment, reflecting real-world clinical practice but necessitating further validation of conclusions regarding high AFP levels and AFP response when ICIs are combined with other therapeutic measures. Third, discrepancies in the definitions of high AFP levels and AFP response across studies, along with the paucity of studies reporting multiple criteria simultaneously, precluded an investigation of different criteria within the same patient groups, potentially introducing some degree of error into the results. Fourth, while some studies conducted dynamic observations of AFP changes, the available datasets were insufficiently large to permit a comprehensive combined analysis.

The rapid expansion of ICIs in the treatment of HCC underscores the importance of promptly ascertaining their efficacy. This can assist in reducing the financial burden undergoing treatment, and facilitate the timely identification of the necessity to modify treatment regimens. The development of predictive scores based on baseline AFP levels in conjunction with post-treatment AFP response, enables the identification of HCC patients suitable for ICIs therapy. The convenience and the relatively low cost of the AFP test render it a relatively accessible option for clinical use. Nevertheless, utilizing AFP to predict the efficacy of ICIs remains a nascent field of study. Therefore, we recommend that future studies explore multiple definitional criteria simultaneously and adopt a dynamic monitoring approach to track changes in AFP levels.

## 5 Conclusion

In ICIs-treated HCC, patients with high AFP levels had shorter OS and PFS and lower DCR. AFP levels were not significantly associated with ORR. AFP responses were associated with improved survival outcomes and disease control. We recommend that future research focus on determining both the optimal cut-off value for high AFP levels and the criteria for early AFP responses to provide an early signal of treatment response before radiological assessment in ICIs-treated HCC, so as to exercise extra caution to assess the benefit-risk ratio in proceeding with subsequent cycles of treatment.

## Data Availability

The original contributions presented in the study are included in the article/**Supplementary Material**. Further inquiries can be directed to the corresponding authors.
