# Supplementary material for: The prognostic and predictive value of AFP in immune checkpoint inhibitor-treated hepatocellular carcinoma: a systematic review and meta-analysis
Source: Front Immunol. 2025 Nov 4;16:1695861. doi: 10.3389/fimmu.2025.1695861 (PMC12623344; doi:10.3389/fimmu.2025.1695861)
Supplement: Supplementary file 1 [file DataSheet1.pdf]

Supplementary Materials

Supplementary Figure 1. A. Sensitivity analysis for high AFP levels OS in ICIs-treated HCC patients; B. Sensitivity analysis for high AFP levels PFS in ICIs-treated HCC patients. 1
Supplementary Figure 2. A. Forest plots of OS subgroup results in ICIs-treated HCC patients with high AFP level; B. Forest plots of PFS subgroup results in ICIs-treated HCC patients with high AFP level (HR>1 means the patients had worse OS or PFS). 2
Supplementary Figure 3. Subgroup results of AFP level OS in ICIs-treated HCC patients; A. Cut-off value subgroups; B. Medication subgroups; C. At least one combination therapy subgroup; D. Two or more combination therapy subgroup; E. Study type subgroups. 3
Supplementary Figure 4. Subgroup results of AFP level PFS in ICIs-treated HCC patients; A. Cut-off value subgroups; B. Medication subgroups; C. At least one combination therapy subgroup; D. Two or more combination therapy subgroup; E. Study type subgroups. 4
Supplementary Figure 5. A. Sensitivity analysis for AFP response OS in ICIs-treated HCC patients; B. Sensitivity analysis for AFP response PFS in ICIs-treated HCC patients. 5
Supplementary Figure 6. A. Forest plots of OS subgroup results in ICIs-treated HCC patients with AFP response; B. Forest plots of PFS subgroup results in ICIs-treated HCC patients with AFP response (HR>1 means the patients had worse OS or PFS). 6
Supplementary Figure 7. Subgroup results of AFP response OS in ICIs-treated HCC patients; A. Decrease degree subgroups; B. Response time subgroups; C. Medication subgroups D. At least one combination therapy subgroup; E. Two or more combination therapy subgroup. 7
Supplementary Figure 8. Subgroup results of AFP response PFS in ICIs-treated HCC patients; A. Decrease degree subgroups; B. Response time subgroups; C. Medication subgroups D. At least one combination therapy subgroup; E. Two or more combination therapy subgroup; F. Study type subgroups. 8
Supplementary Figure 9. A. Forest plots of ORR and DCR subgroup results in ICIs-treated HCC patients with high AFP level; B. Forest plots of ORR and DCR subgroup results in ICIs-treated HCC patients with AFP response (OR>1 means the patients had well ORR or DCR). 9
Supplementary Figure 10. A. Publication bias of AFP level OS in ICIs-treated HCC patients by funnel chart; B. Publication bias of AFP level PFS in ICIs-treated HCC patients by funnel chart. 10
Supplementary Figure 11. A. Publication bias of AFP response OS in ICIs-treated HCC patients by funnel chart; B. Publication bias of AFP response PFS in ICIs-treated HCC patients by funnel chart. 11
Supplementary Table 1. The details information of the treatment measurements of the included studies. 12
Supplementary Table 2. Details of Quality in Prognosis Studies 18
Supplementary Table 3. Results of meta-regression 21
Supplementary Table 4. The trim-and-fill method results of OS. 22
Supplementary Table 5. The trim-and-fill method results of PFS 25
Supplementary Reference 28

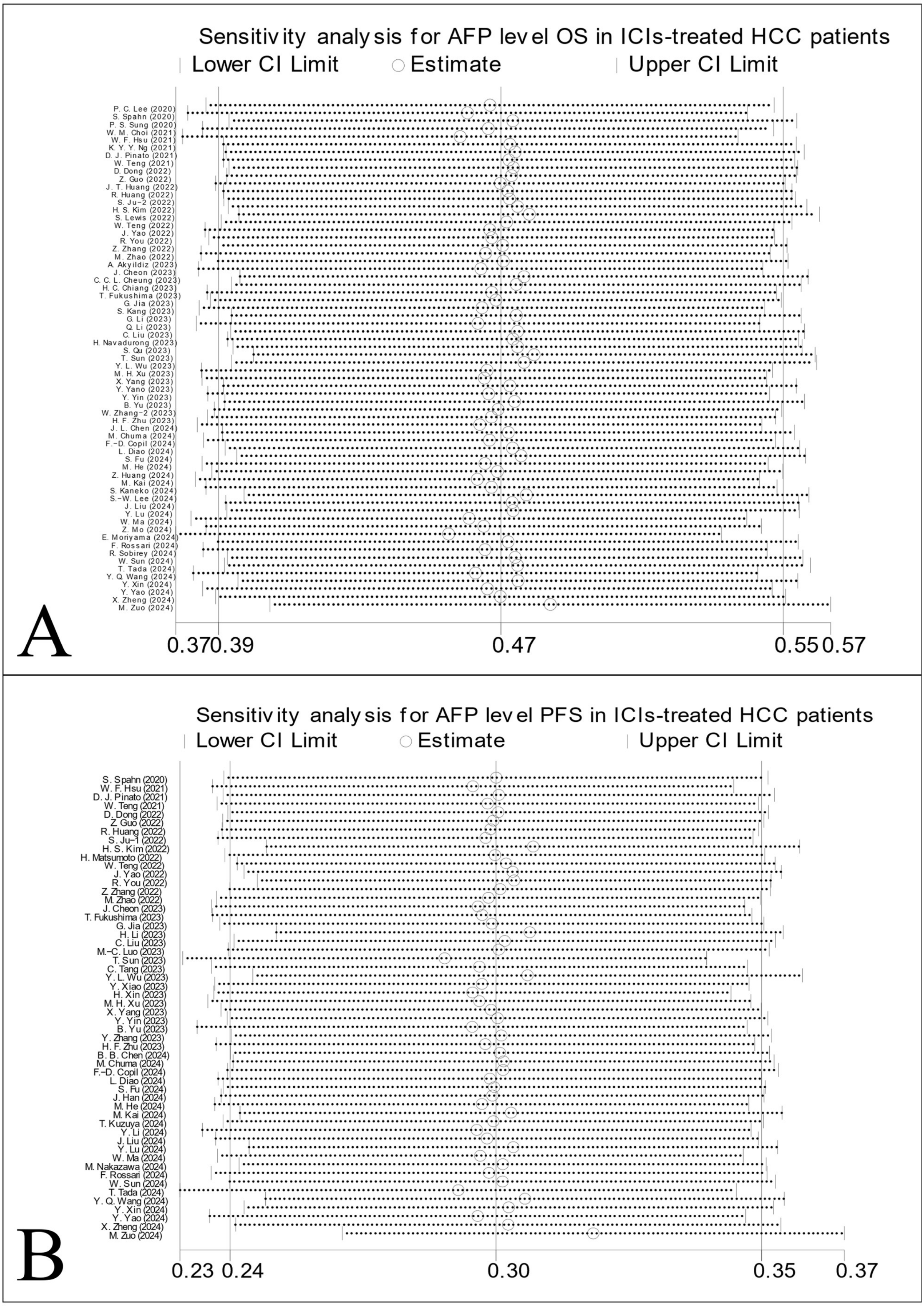

Supplementary Figure 1. A. Sensitivity analysis for high AFP levels OS in ICIs-treated HCC patients; B. Sensitivity analysis for high AFP levels PFS in ICIs-treated HCC patients.

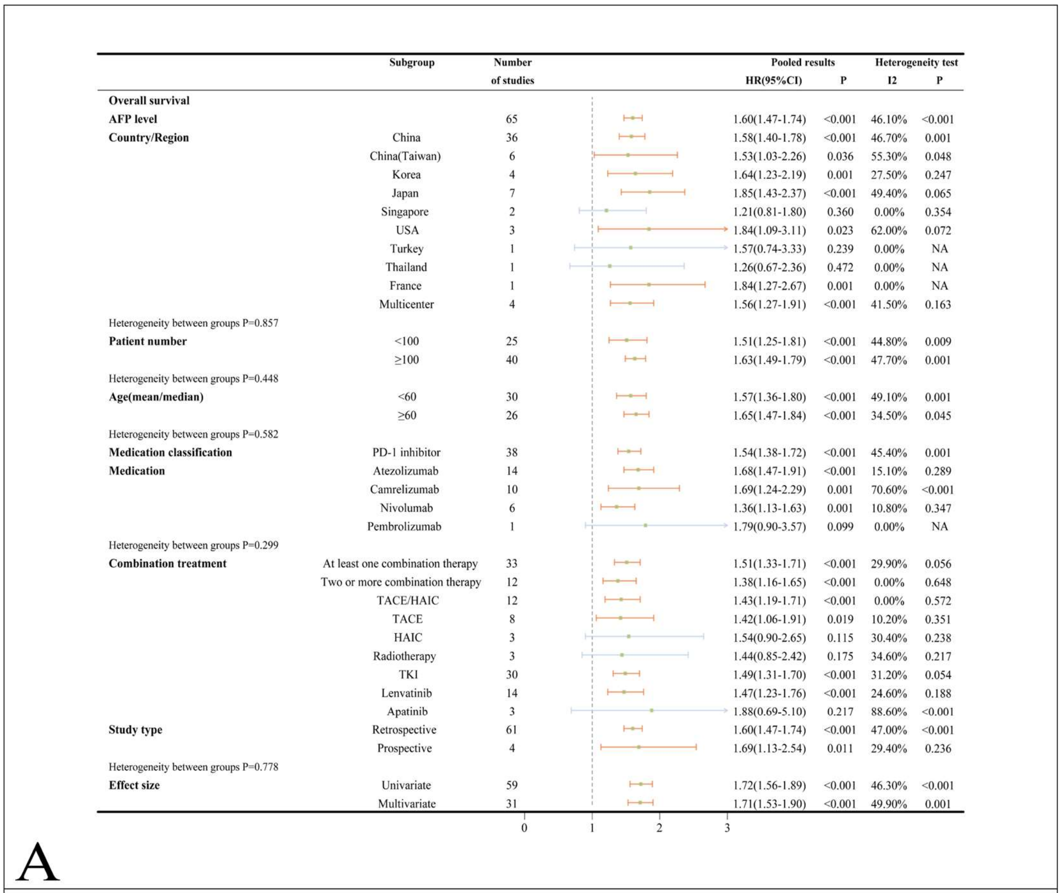

	Subgroup	Number of studies	Pooled results HR(95%CI)	Pooled results P	Heterogeneity test I2	Heterogeneity test P
**Overall survival**						
**AFP level**		65	1.60(1.47-1.74)	<0.001	46.10%	<0.001
**Country/Region**	China	36	1.58(1.40-1.78)	<0.001	46.70%	0.001
	China(Taiwan)	6	1.53(1.03-2.26)	0.036	55.30%	0.048
	Korea	4	1.64(1.23-2.19)	0.001	27.50%	0.247
	Japan	7	1.85(1.43-2.37)	<0.001	49.40%	0.065
	Singapore	2	1.21(0.81-1.80)	0.360	0.00%	0.354
	USA	3	1.84(1.09-3.11)	0.023	62.00%	0.072
	Turkey	1	1.57(0.74-3.33)	0.239	0.00%	NA
	Thailand	1	1.26(0.67-2.36)	0.472	0.00%	NA
	France	1	1.84(1.27-2.67)	0.001	0.00%	NA
	Multicenter	4	1.56(1.27-1.91)	<0.001	41.50%	0.163
Heterogeneity between groups P=0.857						
**Patient number**	<100	25	1.51(1.25-1.81)	<0.001	44.80%	0.009
	≥100	40	1.63(1.49-1.79)	<0.001	47.70%	0.001
Heterogeneity between groups P=0.448						
**Age(mean/median)**	<60	30	1.57(1.36-1.80)	<0.001	49.10%	0.001
	≥60	26	1.65(1.47-1.84)	<0.001	34.50%	0.045
Heterogeneity between groups P=0.582						
**Medication classification**	PD-1 inhibitor	38	1.54(1.38-1.72)	<0.001	45.40%	0.001
**Medication**	Atezolizumab	14	1.68(1.47-1.91)	<0.001	15.10%	0.289
	Camrelizumab	10	1.69(1.24-2.29)	0.001	70.60%	<0.001
	Nivolumab	6	1.36(1.13-1.63)	0.001	10.80%	0.347
	Pembrolizumab	1	1.79(0.90-3.57)	0.099	0.00%	NA
Heterogeneity between groups P=0.299						
**Combination treatment**	At least one combination therapy	33	1.51(1.33-1.71)	<0.001	29.90%	0.056
	Two or more combination therapy	12	1.38(1.16-1.65)	<0.001	0.00%	0.648
	TACE/HAIC	12	1.43(1.19-1.71)	<0.001	0.00%	0.572
	TACE	8	1.42(1.06-1.91)	0.019	10.20%	0.351
	HAIC	3	1.54(0.90-2.65)	0.115	30.40%	0.238
	Radiotherapy	3	1.44(0.85-2.42)	0.175	34.60%	0.217
	TKI	30	1.49(1.31-1.70)	<0.001	31.20%	0.054
	Lenvatinib	14	1.47(1.23-1.76)	<0.001	24.60%	0.188
	Apatinib	3	1.88(0.69-5.10)	0.217	88.60%	<0.001
**Study type**	Retrospective	61	1.60(1.47-1.74)	<0.001	47.00%	<0.001
	Prospective	4	1.69(1.13-2.54)	0.011	29.40%	0.236
Heterogeneity between groups P=0.778						
**Effect size**	Univariate	59	1.72(1.56-1.89)	<0.001	46.30%	<0.001
	Multivariate	31	1.71(1.53-1.90)	<0.001	49.90%	0.001

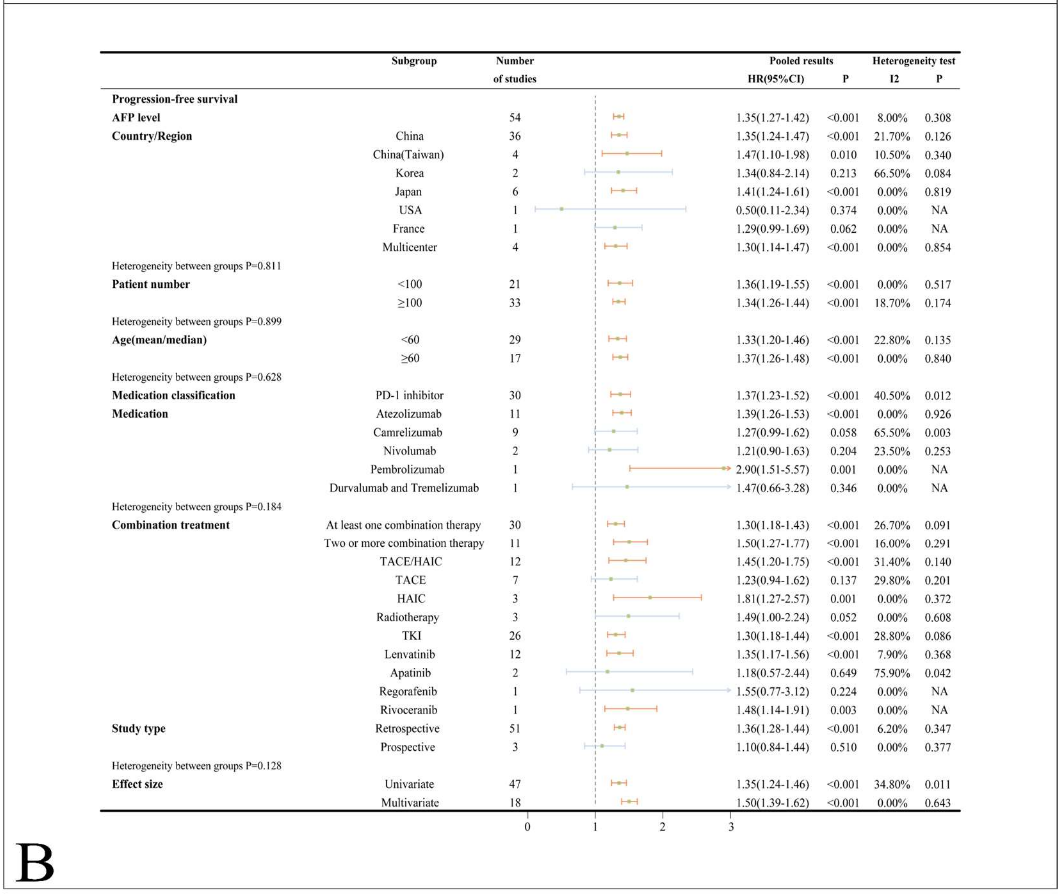

	Subgroup	Number of studies	Pooled results HR(95%CI)	Pooled results P	Heterogeneity test I2	Heterogeneity test P
**Progression-free survival**						
**AFP level**		54	1.35(1.27-1.42)	<0.001	8.00%	0.308
**Country/Region**	China	36	1.35(1.24-1.47)	<0.001	21.70%	0.126
	China(Taiwan)	4	1.47(1.10-1.98)	0.010	10.50%	0.340
	Korea	2	1.34(0.84-2.14)	0.213	66.50%	0.084
	Japan	6	1.41(1.24-1.61)	<0.001	0.00%	0.819
	USA	1	0.50(0.11-2.34)	0.374	0.00%	NA
	France	1	1.29(0.99-1.69)	0.062	0.00%	NA
	Multicenter	4	1.30(1.14-1.47)	<0.001	0.00%	0.854
Heterogeneity between groups P=0.811						
**Patient number**	<100	21	1.36(1.19-1.55)	<0.001	0.00%	0.517
	≥100	33	1.34(1.26-1.44)	<0.001	18.70%	0.174
Heterogeneity between groups P=0.899						
**Age(mean/median)**	<60	29	1.33(1.20-1.46)	<0.001	22.80%	0.135
	≥60	17	1.37(1.26-1.48)	<0.001	0.00%	0.840
Heterogeneity between groups P=0.628						
**Medication classification**	PD-1 inhibitor	30	1.37(1.23-1.52)	<0.001	40.50%	0.012
**Medication**	Atezolizumab	11	1.39(1.26-1.53)	<0.001	0.00%	0.926
	Camrelizumab	9	1.27(0.99-1.62)	0.058	65.50%	0.003
	Nivolumab	2	1.21(0.90-1.63)	0.204	23.50%	0.253
	Pembrolizumab	1	2.90(1.51-5.57)	0.001	0.00%	NA
	Durvalumab and Tremelizumab	1	1.47(0.66-3.28)	0.346	0.00%	NA
Heterogeneity between groups P=0.184						
**Combination treatment**	At least one combination therapy	30	1.30(1.18-1.43)	<0.001	26.70%	0.091
	Two or more combination therapy	11	1.50(1.27-1.77)	<0.001	16.00%	0.291
	TACE/HAIC	12	1.45(1.20-1.75)	<0.001	31.40%	0.140
	TACE	7	1.23(0.94-1.62)	0.137	29.80%	0.201
	HAIC	3	1.81(1.27-2.57)	0.001	0.00%	0.372
	Radiotherapy	3	1.49(1.00-2.24)	0.052	0.00%	0.608
	TKI	26	1.30(1.18-1.44)	<0.001	28.80%	0.086
	Lenvatinib	12	1.35(1.17-1.56)	<0.001	7.90%	0.368
	Apatinib	2	1.18(0.57-2.44)	0.649	75.90%	0.042
	Regorafenib	1	1.55(0.77-3.12)	0.224	0.00%	NA
	Rivoceranib	1	1.48(1.14-1.91)	0.003	0.00%	NA
**Study type**	Retrospective	51	1.36(1.28-1.44)	<0.001	6.20%	0.347
	Prospective	3	1.10(0.84-1.44)	0.510	0.00%	0.377
Heterogeneity between groups P=0.128						
**Effect size**	Univariate	47	1.35(1.24-1.46)	<0.001	34.80%	0.011
	Multivariate	18	1.50(1.39-1.62)	<0.001	0.00%	0.643

Supplementary Figure 2. A. Forest plots of OS subgroup results in ICIs-treated HCC patients with high AFP level; B. Forest plots of PFS subgroup results in ICIs-treated HCC patients with high AFP level (HR>1 means the patients had worse OS or PFS).

OS, overall survival; PFS, progress-free survival; ICI, immune checkpoint inhibitor; HCC, hepatocellular carcinoma; PD-1, programmed cell death 1; TACE, transhepatic arterial chemotherapy and embolization; HAIC, hepatic artery infusion chemotherapy; TKI, tyrosine kinase inhibitor; NA, not available.

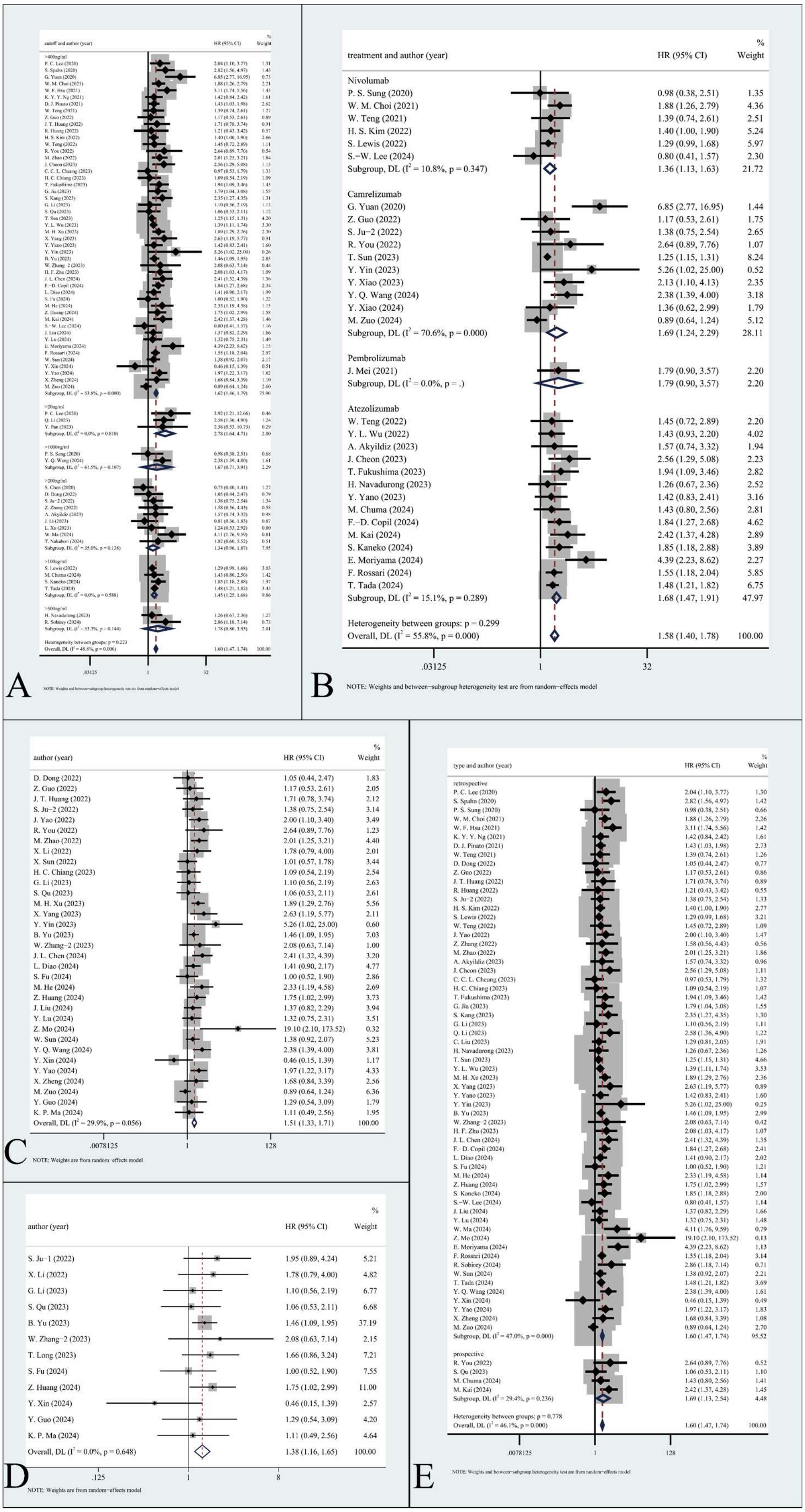

Supplementary Figure 3. Subgroup results of AFP level OS in ICIs-treated HCC patients; A. Cut-off value subgroups; B. Medication subgroups; C. At least one combination therapy subgroup; D. Two or more combination therapy subgroup; E. Study type subgroups.

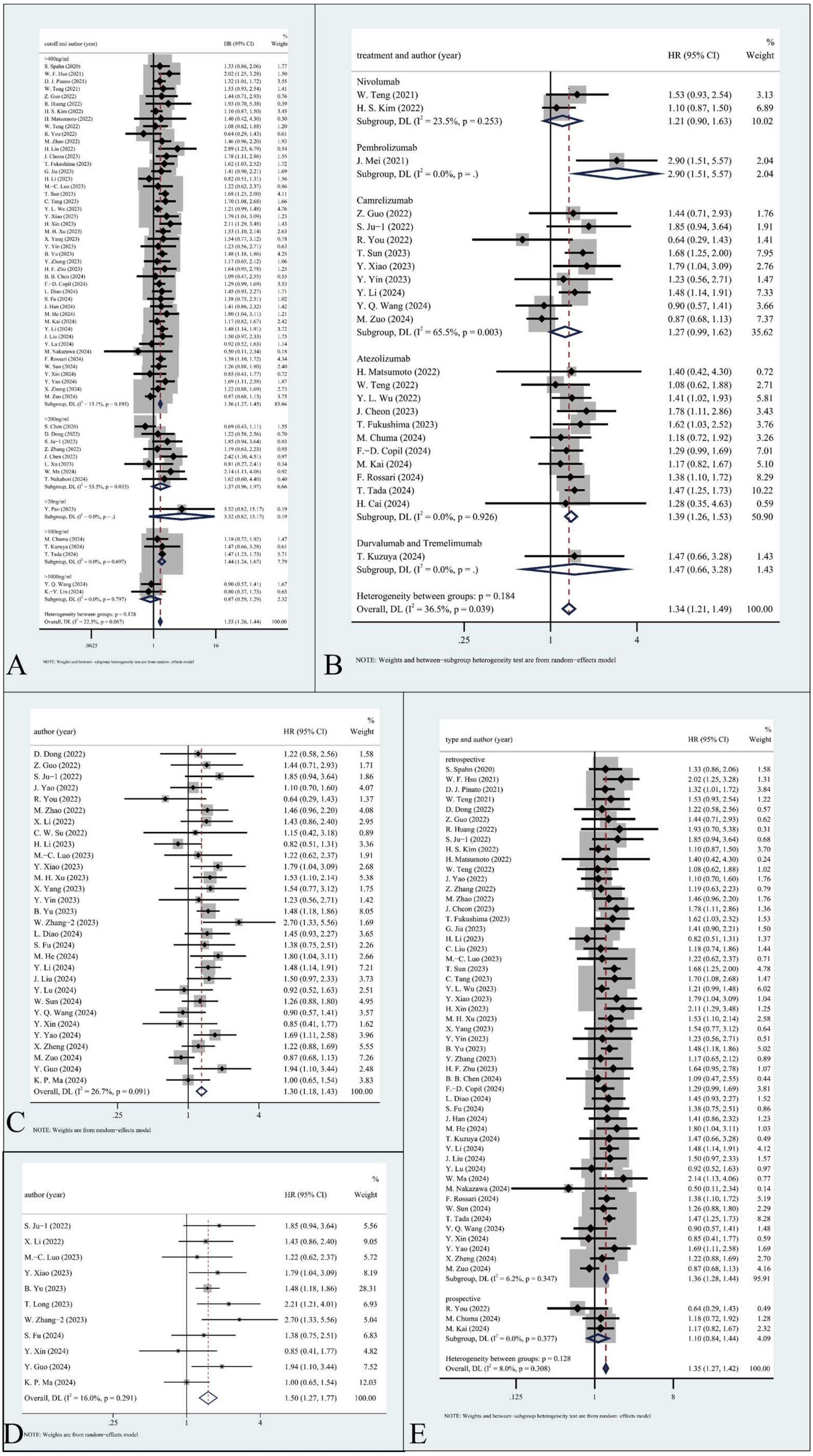

Supplementary Figure 4. Subgroup results of AFP level PFS in ICIs-treated HCC patients; A. Cut-off value subgroups; B. Medication subgroups; C. At least one combination therapy subgroup; D. Two or more combination therapy subgroup; E. Study type subgroups.

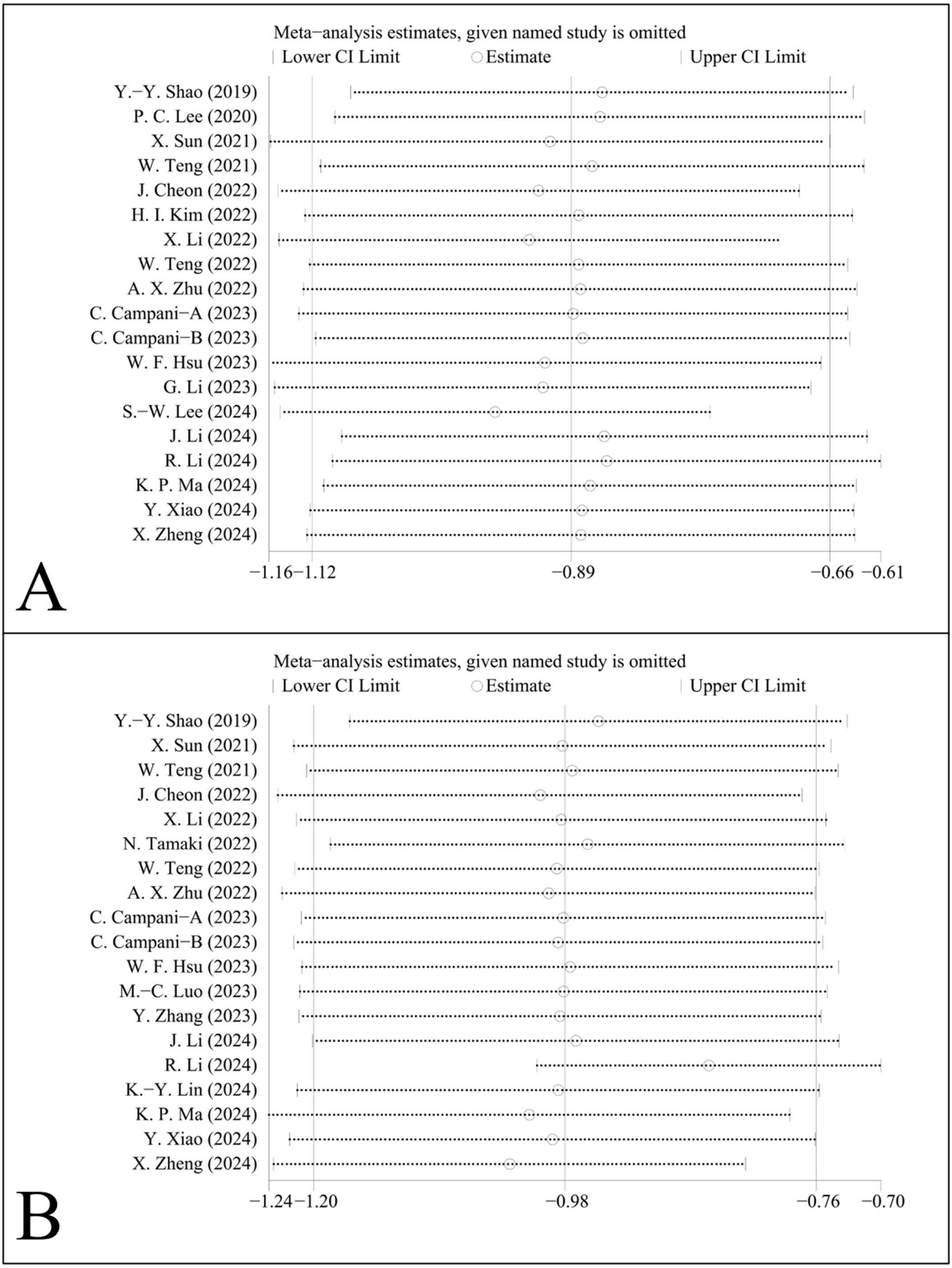

Supplementary Figure 5. A. Sensitivity analysis for AFP response OS in ICIs-treated HCC patients; B. Sensitivity analysis for AFP response PFS in ICIs-treated HCC patients.

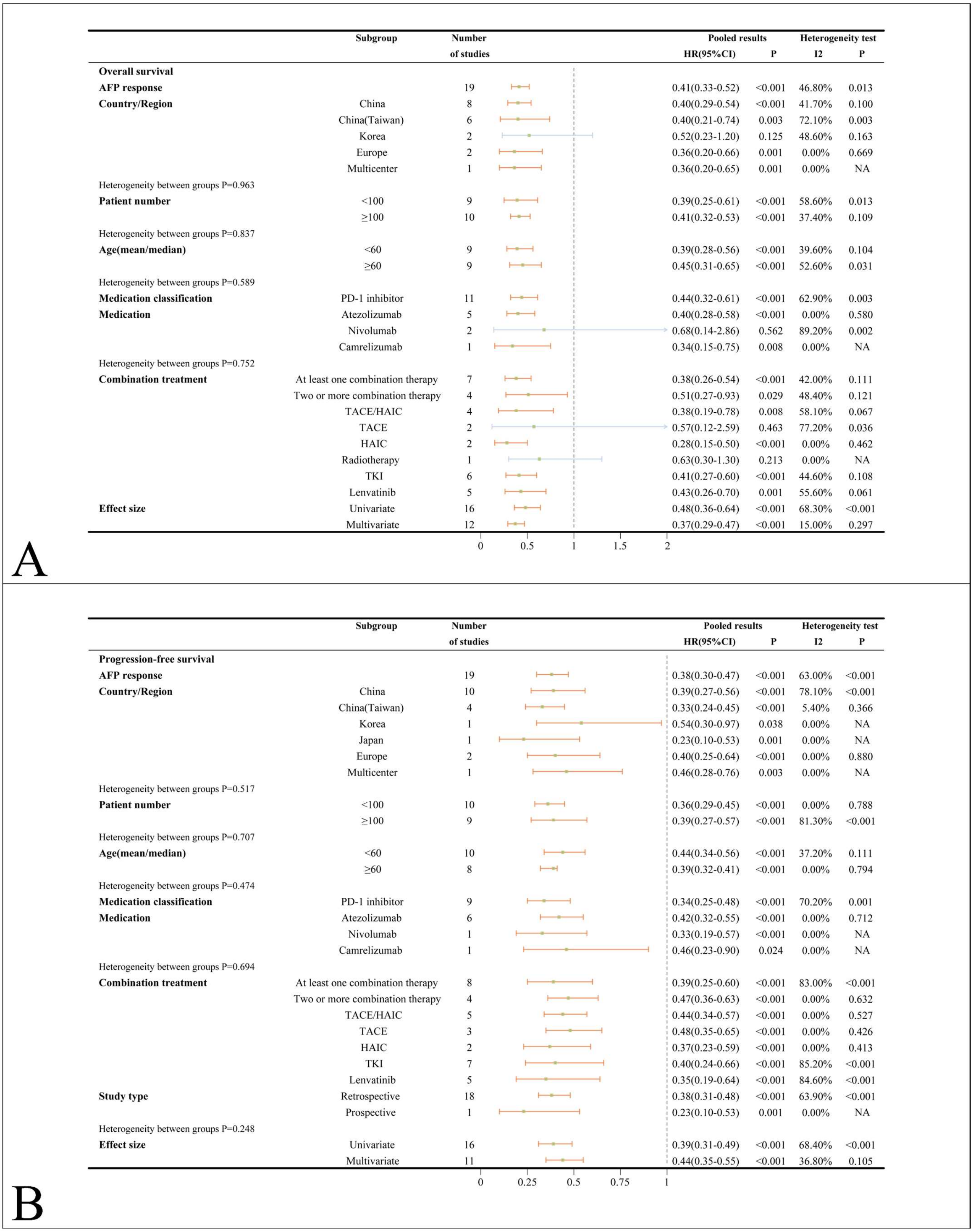

Supplementary Figure 6. A. Forest plots of OS subgroup results in ICIs-treated HCC patients with AFP response; B. Forest plots of PFS subgroup results in ICIs-treated HCC patients with AFP response (HR>1 means the patients had worse OS or PFS).

OS, overall survival; PFS, progress-free survival; ICI, immune checkpoint inhibitor; HCC, hepatocellular carcinoma; PD-1, programmed cell death 1; TACE, transhepatic arterial chemotherapy and embolization; HAIC, hepatic artery infusion chemotherapy; TKI, tyrosine kinase inhibitor; NA, not available.

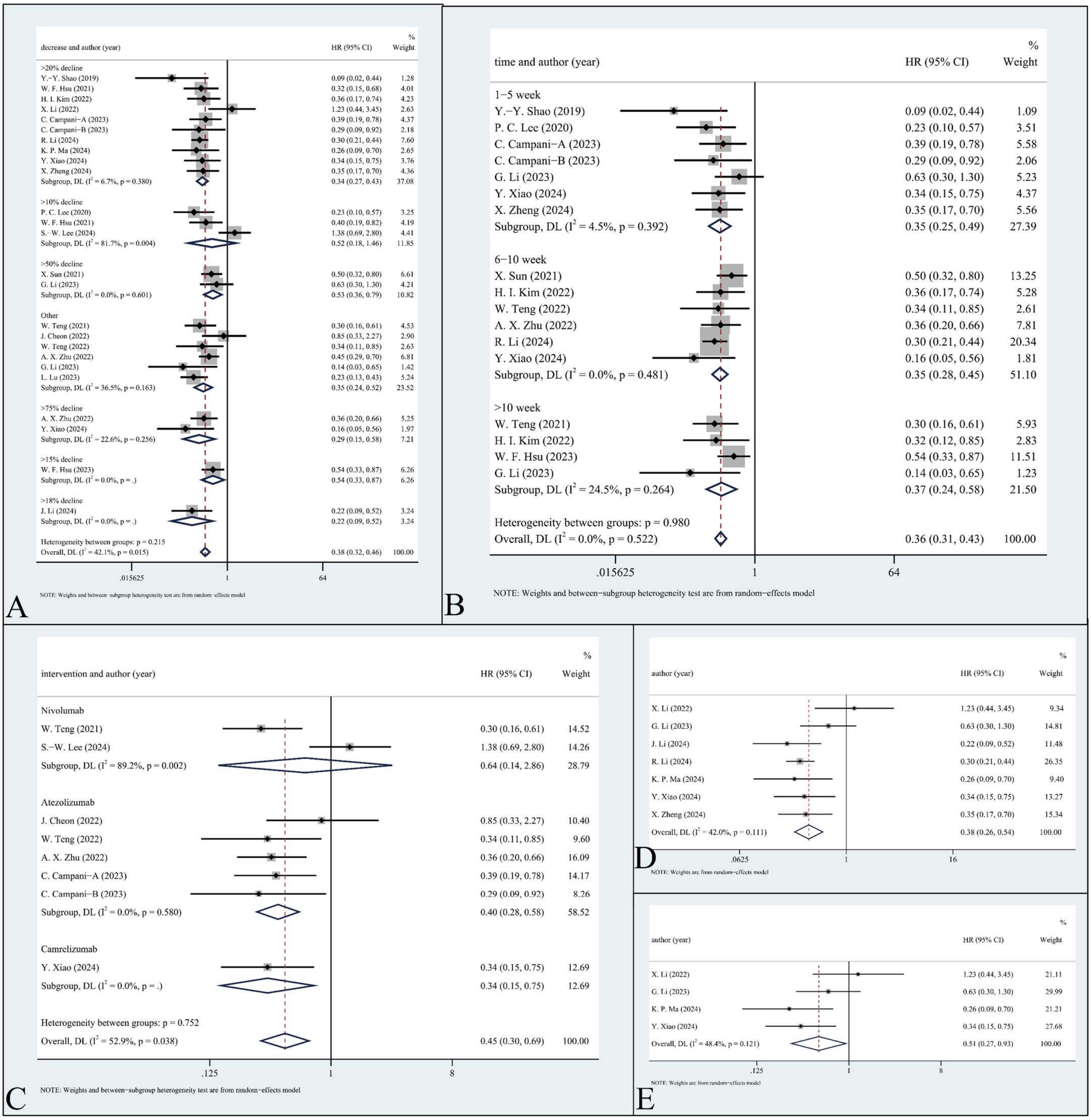

Supplementary Figure 7. Subgroup results of AFP response OS in ICIs-treated HCC patients; A. Decrease degree subgroups; B. Response time subgroups; C. Medication subgroups D. At least one combination therapy subgroup; E. Two or more combination therapy subgroup.

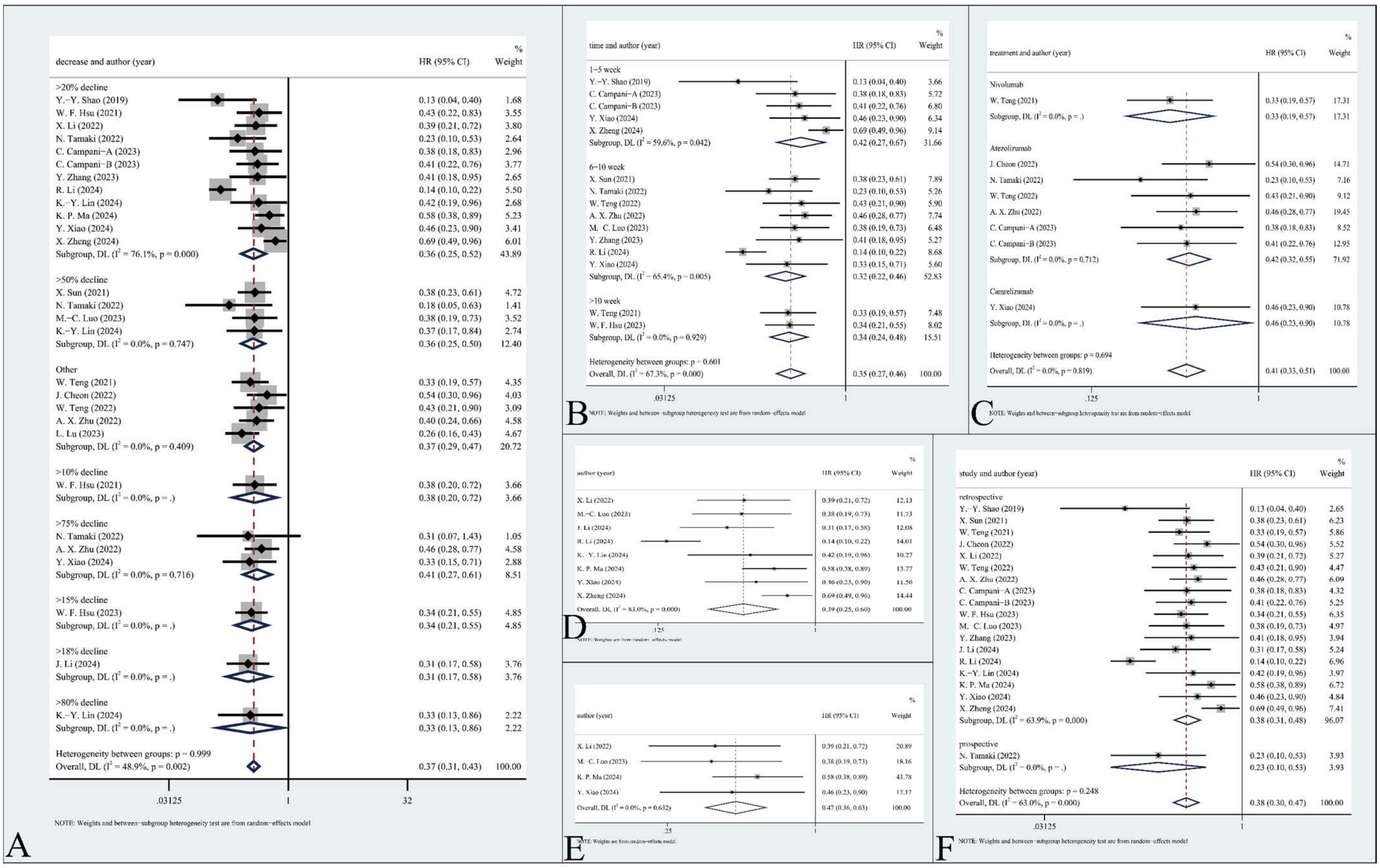

Supplementary Figure 8. Subgroup results of AFP response PFS in ICIs-treated HCC patients; A. Decrease degree subgroups; B. Response time subgroups; C. Medication subgroups D. At least one combination therapy subgroup; E. Two or more combination therapy subgroup; F. Study type subgroups.

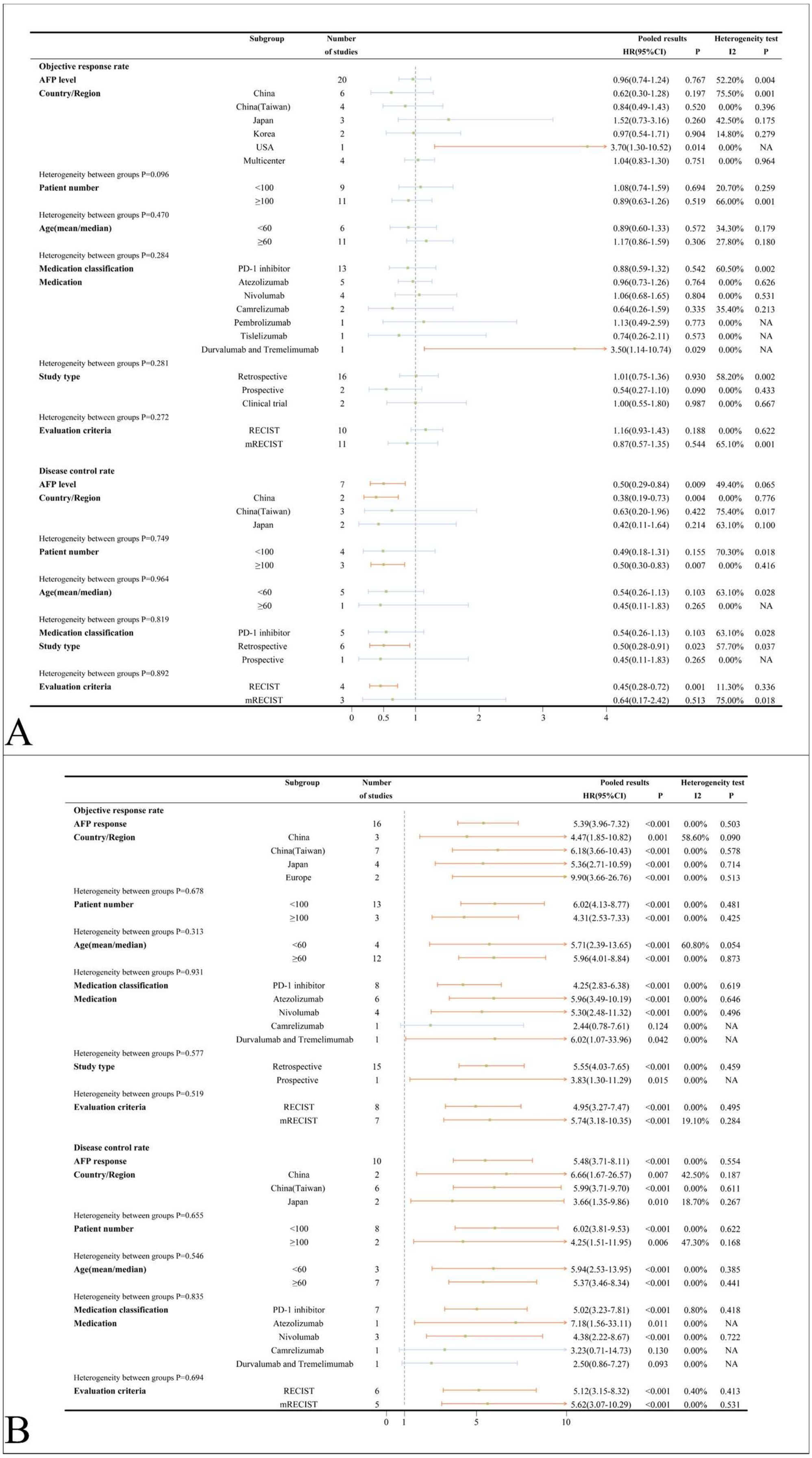

Supplementary Figure 9. A. Forest plots of ORR and DCR subgroup results in ICIs-treated HCC patients with high AFP level; B. Forest plots of ORR and DCR subgroup results in ICIs-treated HCC patients with AFP response (OR>1 means the patients had well ORR or DCR).

ORR, objective response rate; DCR, disease control rate; ICI, immune checkpoint inhibitor; HCC, hepatocellular carcinoma; RECST, Response Evaluation Criteria in Solid Tumors; mRECST, modified Response Evaluation Criteria in Solid Tumors.

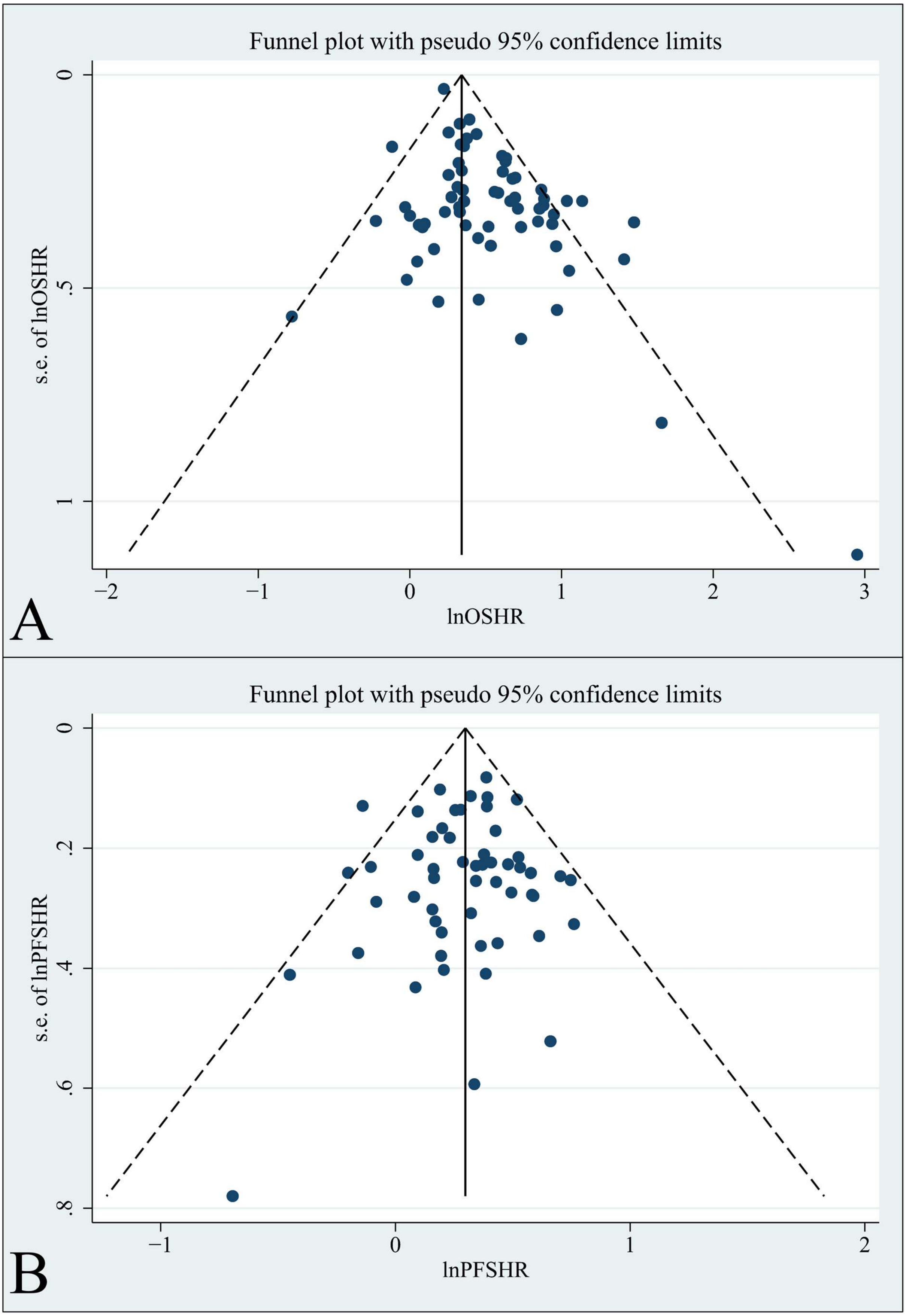

Supplementary Figure 10. A. Publication bias of AFP level OS in ICIs-treated HCC patients by funnel chart; B. Publication bias of AFP level PFS in ICIs-treated HCC patients by funnel chart.

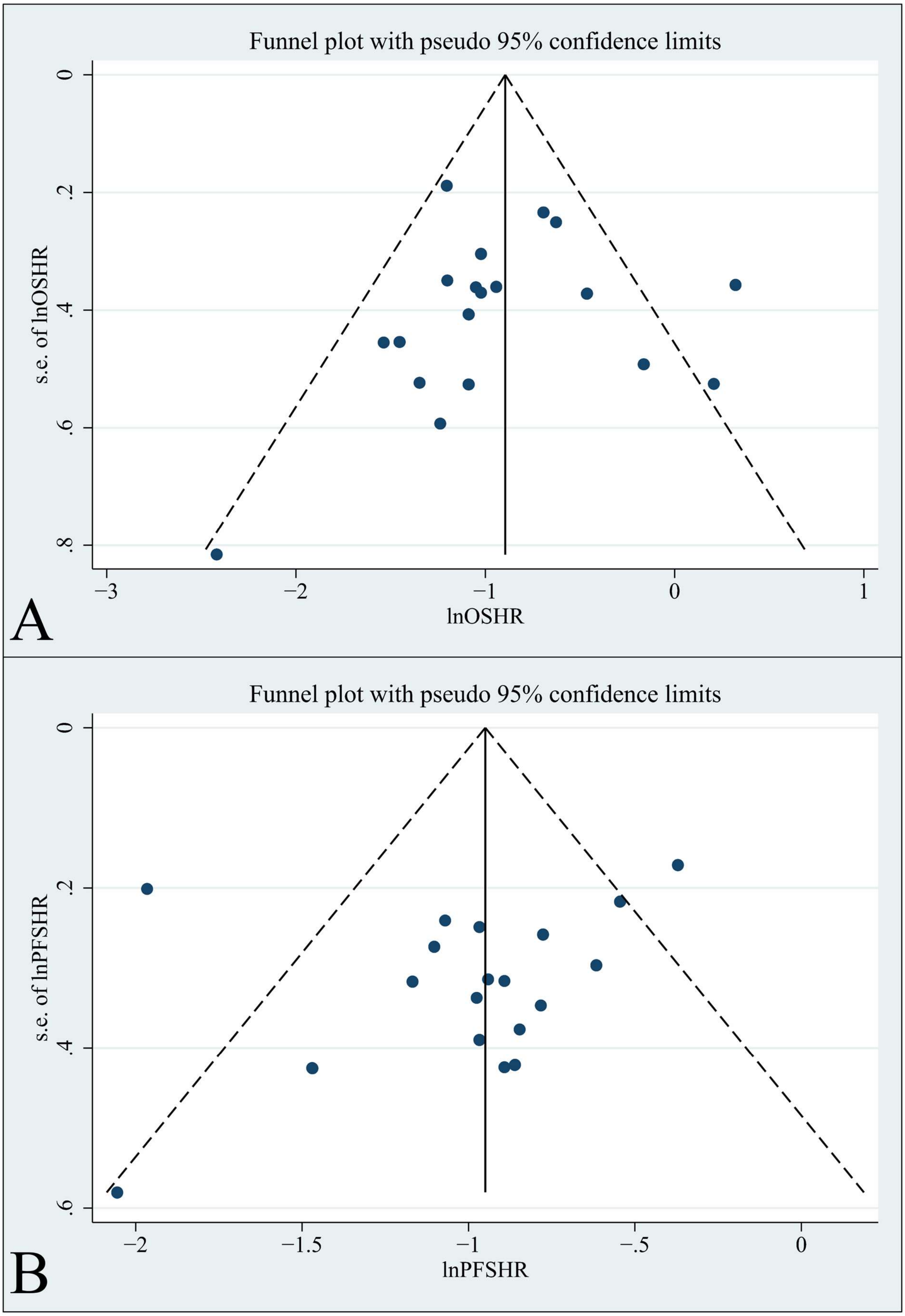

Supplementary Figure 11. A. Publication bias of AFP response OS in ICIs-treated HCC patients by funnel chart; B. Publication bias of AFP response PFS in ICIs-treated HCC patients by funnel chart.

Supplementary Table 1. The details information of the treatment measurements of the included studies.

First author	Year	Intervention	Combination therapy	Previous therapy	Subsequent therapy
Y.-Y. Shao	2019	ICIs	NA	Part received systemic therapy	NA
S. Chen*	2020	PD-1 inhibitors	Part received systemic therapy	NA	NA
W. M. Choi	2020	Nivolumab	NA	Part received systemic therapy	NA
R. S. Finn	2020	Pembrolizumab	Lenvatinib	NA	NA
W. F. Hsu*	2020	Nivolumab	Part received	Part received	NA
M. S. Lee	2020	Atezolizumab	Bevacizumab	None	NA
P. C. Lee*	2020	Nivolumab/Pembrolizumab	Part received	TACE etc	Part received
S. Spahn	2020	Nivolumab/Pembrolizumab	NA	Part received systemic therapy	NA
P. S. Sung	2020	Nivolumab	NA	Part received	Part received
G. Yuan*	2020	Camrelizumab	Apatinib	Part received	NA
W. M. Choi	2021	Nivolumab	NA	NA	NA
W. F. Hsu*	2021	Nivolumab/Pembrolizumab	Part received	Part received	NA
J. Mei*	2021	Pembrolizumab	Lenvatinib, part received HAIC	Part received	NA
M. Morita	2021	PD-1 inhibitors	NA	NA	NA
K. Y. Y. Ng	2021	ICIs	NA	Part received	NA
D. J. Pinato	2021	ICIs	Part received	Part received	NA
X. Sun*	2021	PD-1 inhibitors	Part received	Part received	NA
W. Teng	2021	Nivolumab	Part received	NA	Part received
J. Chen*	2022	PD-1 inhibitors	Part received	NA	NA
S. C. Chen	2022	Nivolumab/Pembrolizumab	Part received sorafenib	NA	NA
J. Cheon*	2022	Atezolizumab	Bevacizumab	Part received	NA
M. Chuma	2022	Atezolizumab	Bevacizumab	Part received	NA

D. Dong	2022	ICIs	RT	Part received	NA
Z. Guo	2022	Camrelizumab	TKIs, part received TACE	NA	NA
Y. Hayakawa	2022	Atezolizumab	Bevacizumab	Part received	NA
J. T. Huang	2022	Camrelizumab/Sintinimab	TKIs, part received TACE	NA	NA
R. Huang	2022	PD-1 inhibitors	NA	Part received	NA
S. Ju-1*	2022	Camrelizumab	Apatinib and TACE	NA	NA
S. Ju-2	2022	Camrelizumab	Apatinib, part received TACE	NA	NA
H. I. Kim	2022	Nivolumab/Atezolizumab	Part received	Part received	NA
H. S. Kim	2022	Nivolumab	NA	Part received	NA
S. W. Lee*	2022	Nivolumab/Pembrolizumab	TKIs	NA	NA
S. Lewis	2022	Nivolumab	NA	Part received	NA
X. Li*	2022	PD-1 inhibitors	Lenvatinib and TACE	Part received	Part received
H. Liu*	2022	Camrelizumab	NA	Part received	NA
H. Matsumoto	2022	Atezolizumab	Bevacizumab	NA	NA
T.-R. Peng	2022	Nivolumab	Sorafenib	NA	NA
C. W. Su*	2022	ICIs	Proton beam radiotherapy	Part received	NA
X. Sun*	2022	PD-1 inhibitors	Lenvatinib	Part received	NA
N. Tamaki	2022	Atezolizumab	Bevacizumab	Part received	NA
W. Teng	2022	Atezolizumab	Bevacizumab	Part received	NA
Y. L. Wu*	2022	Atezolizumab	Bevacizumab	Part received	NA
Y. J. Xiang*	2022	Toripalimab/Sintilimab	TACE, part received anti-angiogenic therapy	NA	NA
J. Yao	2022	PD-1 inhibitors	Antiangiogenic therapy	Part received	NA
R. You	2022	Camrelizumab	TACE	Part received	NA
Z. Zhang	2022	PD-1 inhibitors	NA	Part received	NA

M. Zhao	2022	ICIs	TKIs	NA	NA
A. X. Zhu	2022	Atezolizumab	Bevacizumab	Part received	NA
A. Akyildiz	2023	Atezolizumab	Bevacizumab	Part received	NA
C. Campani-A	2023	Atezolizumab	Bevacizumab	Part received systemic therapy	NA
C. Campani-B	2023	Atezolizumab	Bevacizumab	Part received systemic therapy	NA
J. Cheon	2023	Atezolizumab	Bevacizumab	Part received	NA
C. C. L. Cheung	2023	ICIs	NA	Part received	NA
H. C. Chiang	2023	Nivolumab/Pembrolizumab	Lenvatinib/Sorafenib	Part received	NA
T. Fukushima	2023	Atezolizumab	Bevacizumab	Part received	NA
C. Hong	2023	ICIs	Part received targeted drugs	Part received	NA
W. F. Hsu	2023	Nivolumab/Pembrolizumab	Part received	Part received	NA
G. Jia	2023	PD-1 inhibitors	Part received	Part received	NA
S. Kang	2023	ICIs	Part received	Part received	NA
G. Li	2023	PD-1 inhibitors	Lenvatinib and radiotherapy	Part received	NA
H. Li*	2023	PD-1 inhibitors	Antiangiogenic therapy, part received TACE	NA	NA
J. Li*	2023	Pembrolizumab/Sintilimab	Part received	Part received	NA
Q. Li	2023	PD-1 inhibitors	NA	Part received	NA
S. Li	2023	PD-1 inhibitors	Lenvatinib and TACE	NA	NA
C. Liu	2023	ICIs	NA	Part received	NA
T. Long*	2023	PD-1 inhibitors	TKIs and TACE/HAIC	NA	NA
L. Lu*	2023	Atezolizumab	Bevacizumab	Part received	NA
M.-C. Luo*	2023	ICIs	Lenvatinib and TACE	NA	NA
H. Navadurong	2023	Atezolizumab	Bevacizumab	Part received	NA
Y. Pan*	2023	PD-1 inhibitors	TKIs	Part received	Hepatectomy

M. Persano*	2023	Atezolizumab	Bevacizumab	Part received	NA
S. Qu	2023	PD-1 inhibitors	Lenvatinib and TACE	NA	NA
R. Raj	2023	ICIs	Part received	Part received	NA
T. Sun	2023	Camrelizumab	Part received	NA	NA
N. Tanabe	2023	Atezolizumab	Bevacizumab	Part received	NA
C. Tang	2023	ICIs	Bevacizumab, part received radiotherapy	NA	NA
J. Wang*	2023	PD-1 inhibitors	Lenvatinib, part received TACE	Part received	NA
Y. L. Wu	2023	ICIs	Part received	Part received	NA
Y. Xiao*	2023	Camrelizumab	Lenvatinib and HAIC	NA	NA
H. Xin	2023	ICIs	NA	NA	NA
L. Xu*	2023	PD-1 inhibitors	NA	Part received	Part received
M. H. Xu	2023	PD-1 inhibitors	Lenvatinib	Part received	NA
X. Yang	2023	ICIs	Regorafenib, part received TACE	Part received	NA
Y. Yano	2023	Atezolizumab	Bevacizumab	Part received	NA
Y. Yin	2023	Camrelizumab	TKIs, part received TACE	Part received	NA
B. Yu	2023	PD-1 inhibitors	TKIs, HAIC or TACE	NA	NA
W. Zhang-1*	2023	PD-1 inhibitors	Lenvatinib	Part received	Part received
W. Zhang-2*	2023	PD-1 inhibitors	Targeted therapy and HAIC	NA	NA
Y. Zhang	2023	ICIs	Part received	NA	NA
H. F. Zhu	2023	ICIs	Part received	NA	NA
H. Cai*	2024	Atezolizumab	Bevacizumab and TAE and HAIC	NA	NA
B. B. Chen	2024	ICIs	Part received	NA	NA
J. L. Chen	2024	ICIs	Targeted therapy	NA	NA
Y. Chen*	2024	PD-1 inhibitors	TKIs	NA	NA

M. Chuma	2024	Atezolizumab	Bevacizumab	NA	NA
F.-D. Copil	2024	Atezolizumab	Bevacizumab	Part received	NA
L. Diao	2024	ICIs	Lenvatinib, part received HAIC	NA	NA
S. Fu	2024	PD-1 inhibitors	Lenvatinib and HAIC	NA	Part received
Y. Guo*	2024	Camrelizumab	TKIs and TACE	NA	NA
J. Han	2024	ICIs	Part received	NA	NA
M. He	2024	PD-1 inhibitors	IMRT	Part received	NA
Z. Huang	2024	PD-1 inhibitors	TKIs and TACE, part received HAIC	NA	NA
M. Kai	2024	Atezolizumab	Bevacizumab	Part received	NA
S. Kaneko	2024	Atezolizumab	Bevacizumab	NA	NA
T. Kuzuya	2024	Durvalumab and Tremelimumab	NA	NA	NA
S.-W. Lee	2024	Nivolumab	Part received	NA	NA
J. Li	2024	PD-1 inhibitors	HAIC	Part received	NA
R. Li*	2024	PD-1 inhibitors	Lenvatinib, part received HAIC	NA	NA
Y. Li	2024	Camrelizumab	Rivoceranib, part received HAIC	NA	NA
K.-Y. Lin*	2024	PD-1 inhibitors	TKIs	NA	Salvage hepatectomy
J. Liu	2024	ICIs	Targeted therapy, part received locoregional	Part received	NA
Y. Lu	2024	ICIs	Kinase inhibitors	Part received	NA
K. P. Ma*	2024	PD-1 inhibitors	TACE and lenvatinib	NA	NA
W. Ma	2024	ICIs	Part received	NA	NA
Z. Mo	2024	ICIs	Angiogenesis blockade therapy	NA	NA
E. Moriyama	2024	Atezolizumab	Bevacizumab	NA	Part received TACE
T. Nakabori*	2024	Atezolizumab	Bevacizumab, part received locoregional	NA	NA
M. Nakazawa	2024	ICIs	Part received	NA	Liver resection

F. Rossari	2024	Atezolizumab	Bevacizumab	Part received	NA
I. Saeki	2024	Durvalumab and Tremelimumab	Part received	NA	NA
R. Sobirey	2024	ICIs	NA	Part received	NA
W. Sun	2024	PD-1 inhibitors	Lenvatinib	Part received	NA
T. Tada	2024	Atezolizumab	Bevacizumab	Part received	Part received
L. Wang*	2024	PD-1 inhibitors	TKIs and locoregional treatment	NA	Part received hepatectomy
Y. Q. Wang	2024	Camrelizumab	Lenvatinib	NA	NA
Y. Xiao*	2024	Camrelizumab	HAIC and lenvatinib	NA	NA
Y. Xin	2024	PD-1 inhibitors	Lenvatinib and TACE	NA	NA
L. Xu	2024	Tislelizumab	Lenvatinib	Part received	NA
J. Yang*	2024	ICIs	Targeted therapy	NA	NA
Y. Yao	2024	PD-1 inhibitors	Lenvatinib	NA	NA
X. Zheng	2024	ICIs	TKIs	Part received TACE	Part received TACE
M. Zuo	2024	Camrelizumab	Apatinib, part received HAIC	NA	NA

ICI, immune checkpoint inhibitor; PD-1, programmed cell death 1; TACE, transhepatic arterial chemotherapy and embolization; HAIC, hepatic artery infusion chemotherapy; TKI, tyrosine kinase inhibitor; TAE, transarterial embolization; IMRT, intensity-modulated radiotherapy; NA, not available.

*: Studies were only partially included (e.g. only used as partial subgroup analyses) due to partial duplication of populations with other studies that were included in the analysis.

Supplementary Table 2. Details of Quality in Prognosis Studies.

First author	year	Study Participation	Study Attrition	Prognostic Factor Measurement	Outcome Measurement	Study Confounding	Statistical Analysis and Reporting
Y.-Y. Shao	2019	Moderate	Low	Low	Low	Low	Moderate
S. Chen*	2020	Moderate	Low	Low	Low	High	Moderate
W. M. Choi	2020	Low	Moderate	Low	Low	High	Moderate
R. S. Finn	2020	Low	Low	Low	Low	High	Low
W. F. Hsu*	2020	Moderate	Moderate	Low	Low	Moderate	Moderate
M. S. Lee	2020	Low	Low	Low	Low	High	Low
P. C. Lee*	2020	Low	Low	Low	Low	Low	Moderate
S. Spahn	2020	Low	Low	Low	Low	High	Moderate
P. S. Sung	2020	High	Moderate	Low	Low	High	Moderate
G. Yuan*	2020	Moderate	Low	Low	Low	Moderate	Moderate
W. M. Choi	2021	Low	Low	Low	Low	Low	Moderate
W. F. Hsu*	2021	Low	Moderate	Low	Low	Moderate	Moderate
J. Mei*	2021	Low	Moderate	Low	Low	Moderate	Moderate
M. Morita	2021	Moderate	Moderate	Low	Low	High	Moderate
K. Y. Y. Ng	2021	Low	Moderate	Low	Low	High	Moderate
D. J. Pinato	2021	Low	Low	Low	Low	Low	Low
X. Sun*	2021	Low	Low	Low	Low	Low	Moderate
W. Teng	2021	Low	Moderate	Low	Low	Moderate	Moderate
J. Chen*	2022	Low	Moderate	Low	Low	Low	Moderate
S. C. Chen	2022	Low	Moderate	Low	Low	High	Moderate
J. Cheon*	2022	Low	Moderate	Low	Low	Low	Low
M. Chuma	2022	Low	Low	Low	Low	High	Moderate
D. Dong	2022	Moderate	Moderate	Low	Low	High	Moderate
Z. Guo	2022	Moderate	High	Low	Low	High	Moderate
Y. Hayakawa	2022	Moderate	High	Low	Low	High	Moderate
J. T. Huang	2022	Moderate	Moderate	Low	Low	High	Moderate
R. Huang	2022	Low	Moderate	Low	Low	Low	Moderate
S. Ju-1*	2022	Low	Low	Low	Low	High	Moderate
S. Ju-2	2022	Low	Low	Low	Low	High	Moderate
H. I. Kim	2022	Low	Low	Low	Low	Low	Moderate
H. S. Kim	2022	Low	Moderate	Low	Low	High	Low
S. W. Lee*	2022	Moderate	Moderate	Low	Low	High	Moderate
S. Lewis	2022	Moderate	Low	Low	Low	High	Moderate
X. Li*	2022	Low	Low	Low	Low	High	Moderate
H. Liu*	2022	Moderate	Low	Low	Low	Moderate	Moderate
H. Matsumoto	2022	Moderate	Moderate	Low	Low	High	Moderate
T.-R. Peng	2022	Moderate	Low	Low	Low	High	Moderate
C. W. Su*	2022	High	Moderate	Low	Low	High	Moderate
X. Sun*	2022	Low	Moderate	Low	Low	High	Moderate
N. Tamaki	2022	Low	Low	Low	Low	High	Low
W. Teng	2022	Low	Low	Low	Low	Moderate	Moderate
Y. L. Wu*	2022	Low	Moderate	Low	Low	Low	Low
Y. J. Xiang*	2022	Low	Low	Low	Low	High	Moderate

J. Yao	2022	Low	Low	Low	Low	Low	Moderate
R. You	2022	Low	Low	Low	Low	Low	Moderate
Z. Zhang	2022	Low	Low	Low	Low	High	Moderate
M. Zhao	2022	Low	Low	Low	Low	Low	Moderate
A. X. Zhu	2022	Low	Low	Low	Low	High	Low
A. Akyildiz	2023	Low	Moderate	Low	Low	High	Low
C. Campani-A	2023	Moderate	Low	Low	Low	High	Moderate
C. Campani-B	2023	Moderate	Low	Low	Low	High	Moderate
J. Cheon	2023	Low	Moderate	Low	Low	Low	Moderate
C. C. L. Cheung	2023	Moderate	Moderate	Low	Low	High	Moderate
H. C. Chiang	2023	Low	Low	Low	Low	Low	Moderate
T. Fukushima	2023	Low	Moderate	Low	Low	Low	Low
C. Hong	2023	Low	Low	Low	Low	Low	Moderate
W. F. Hsu	2023	Low	Low	Low	Low	High	Moderate
G. Jia	2023	Low	Moderate	Low	Low	Low	Moderate
S. Kang	2023	Low	Moderate	Low	Low	High	Moderate
G. Li	2023	Moderate	Low	Low	Low	Moderate	Moderate
H. Li*	2023	Low	Moderate	Low	Low	Moderate	Moderate
J. Li*	2023	Low	Moderate	Low	Low	Low	Moderate
Q. Li	2023	Low	Moderate	Low	Low	Moderate	Moderate
S. Li	2023	Low	Moderate	Low	Low	High	Moderate
C. Liu	2023	Low	Moderate	Low	Low	High	Moderate
T. Long*	2023	Low	Low	Low	Low	Moderate	Moderate
L. Lu*	2023	Low	Low	Low	Low	Low	Low
M.-C. Luo*	2023	Low	Low	Low	Low	Moderate	Moderate
H. Navadurong	2023	Moderate	Low	Low	Low	High	Moderate
Y. Pan*	2023	Moderate	Moderate	Low	Low	High	Moderate
M. Persano*	2023	Low	Low	Low	Low	High	Low
S. Qu	2023	Low	Low	Low	Low	High	Low
R. Raj	2023	Low	Moderate	Low	Low	Moderate	Moderate
T. Sun	2023	Low	Moderate	Low	Low	Low	Moderate
N. Tanabe	2023	Low	Low	Low	Low	Low	Moderate
C. Tang	2023	Low	Moderate	Low	Low	Low	Moderate
J. Wang*	2023	Low	Low	Low	Low	Moderate	Moderate
Y. L. Wu	2023	Low	Low	Low	Low	Moderate	Low
Y. Xiao*	2023	Low	Moderate	Low	Low	Moderate	Moderate
H. Xin	2023	Low	Low	Low	Low	Moderate	Moderate
L. Xu*	2023	Low	Moderate	Low	Low	High	Moderate
M. H. Xu	2023	Low	Low	Low	Low	High	Moderate
X. Yang	2023	Moderate	Low	Low	Low	Moderate	Moderate
Y. Yano	2023	Low	Moderate	Low	Low	High	Low
Y. Yin	2023	Moderate	Low	Low	Low	Low	Moderate
B. Yu	2023	Low	Low	Low	Low	Moderate	Moderate
W. Zhang-1*	2023	Low	Low	Low	Low	Moderate	Moderate
W. Zhang-2*	2023	Low	Low	Low	Low	High	Moderate
Y. Zhang	2023	Low	Low	Low	Low	Low	Moderate
H. F. Zhu	2023	Low	Moderate	Low	Low	Moderate	Moderate

H. Cai*	2024	High	Low	Low	Low	Moderate	Moderate
B. B. Chen	2024	Low	Low	Low	Low	Low	Moderate
J. L. Chen	2024	Low	Moderate	Low	Low	Low	Moderate
Y. Chen*	2024	Moderate	Moderate	Low	Low	High	Moderate
M. Chuma	2024	Low	Low	Low	Low	High	Low
F.-D. Copil	2024	Low	Moderate	Low	Low	Low	Low
L. Diao	2024	Low	Low	Low	Low	High	Moderate
S. Fu	2024	Low	Low	Low	Low	High	Moderate
Y. Guo*	2024	Low	Moderate	Low	Low	Moderate	Moderate
J. Han	2024	Low	Low	Low	Low	Moderate	Moderate
M. He	2024	Low	Low	Low	Low	Low	Moderate
Z. Huang	2024	Low	Low	Low	Low	Low	Moderate
M. Kai	2024	Low	Low	Low	Low	Low	Low
S. Kaneko	2024	Low	Low	Low	Low	Moderate	Low
T. Kuzuya	2024	Moderate	Moderate	Low	Low	High	Moderate
S.-W. Lee	2024	Moderate	Moderate	Low	Low	Moderate	Moderate
J. Li	2024	Low	Moderate	Low	Low	Low	Moderate
R. Li*	2024	Low	Moderate	Low	Low	High	Low
Y. Li	2024	Low	Low	Low	Low	Low	Low
K.-Y. Lin*	2024	Low	Low	Low	Low	Low	Low
J. Liu	2024	Low	Moderate	Low	Low	High	Moderate
Y. Lu	2024	Low	Moderate	Low	Low	Low	Moderate
K. P. Ma*	2024	Low	Low	Low	Low	Moderate	Moderate
W. Ma	2024	High	Moderate	Low	Low	High	Moderate
Z. Mo	2024	Low	Moderate	Low	Low	Low	Moderate
E. Moriyama	2024	Low	Low	Low	Low	High	Moderate
T. Nakabori*	2024	High	Moderate	Low	Low	High	Moderate
M. Nakazawa	2024	High	Moderate	Low	Low	High	Moderate
F. Rossari	2024	Low	Low	Low	Low	Low	Low
I. Saeki	2024	Low	Low	Low	Low	Moderate	Moderate
R. Sobirey	2024	High	Low	Low	Low	High	Moderate
W. Sun	2024	Low	Moderate	Low	Low	Low	Moderate
T. Tada	2024	Low	Low	Low	Low	Low	Low
L. Wang*	2024	Low	Moderate	Low	Low	Moderate	Moderate
Y. Q. Wang	2024	Low	Low	Low	Low	Low	Moderate
Y. Xiao*	2024	Low	Moderate	Low	Low	Moderate	Moderate
Y. Xin	2024	Moderate	Moderate	Low	Low	High	Moderate
L. Xu	2024	Low	Low	Low	Low	High	Low
J. Yang*	2024	Moderate	Moderate	Low	Low	Moderate	Moderate
Y. Yao	2024	Low	Moderate	Low	Low	Low	Moderate
X. Zheng	2024	Low	Low	Low	Low	Low	Moderate
M. Zuo	2024	Low	Low	Low	Low	High	Moderate

Supplementary Table 3. Results of meta-regression

			Meta-Regression	
**Index**	**Covariates**	**Number**	**P-value**	**Adj R-squared**
**AFP response-OS**	**Decrease degree**	25	0.283	6.52%
	**Response time**	17	0.699	-41.30%
	**Country/Region**	19	0.918	-18.93%
	**Patient number**	19	0.907	-19.22%
	**Age(mean/median)**	18	0.595	-16.67%
	**Medication**	8	0.436	-7.37%
**AFP level-OS**	**AFP cutoff**	69	0.561	-7.74%
	**Country/Region**	65	0.978	-6.94%
	**Patient number**	65	0.377	-9.69%
	**Age(mean/median)**	56	0.479	-0.35%
	**Medication**	31	0.209	7.39%
	**Study type**	65	0.759	-2.11%
**AFP response-PFS**	**Decrease degree**	28	0.885	-6.00%
	**Response time**	15	0.400	4.15%
	**Country/Region**	19	0.706	-6.42%
	**Patient number**	19	0.642	-6.51%
	**Age(mean/median)**	18	0.362	20.79%
	**Medication**	8	0.528	0.00%
	**Study type**	19	0.365	-0.31%
**AFP level-PFS**	**AFP cutoff**	59	0.381	-18.80%
	**Medication**	24	0.673	-20.71%
	**Country/Region**	54	0.590	-9.62%
	**Patient number**	54	0.914	-5.92%
	**Age(mean/median)**	46	0.640	-9.83%
	**Study type**	54	0.151	0.04%

AFP, Alpha-fetoprotein; OS, overall survival; PFS, progression-free survival

Supplementary Table 4. The trim-and-fill method results of OS.

Index	Subgroup	Study number	HR (95%CI)	publication bias test P(egger)	Number of additional studies for MetaTrim	Filled HR (95% CI)
**OS**						
**AFP response**		19	0.41(0.33-0.52)	0.795	4	0.46(0.37-0.58)
**Decrease degree**	>20% decline	10	0.34(0.27-0.43)	0.909	0	0.34(0.27-0.43)
	>10% decline	3	0.52(0.18-1.46)	NA	0	0.52(0.18-1.46)
	>50% decline	2	0.53(0.36-0.79)	NA	1	0.50(0.36-0.70)
	>75% decline	2	0.29(0.15-0.58)	NA	1	0.36(0.20-0.64)
	Other	6	0.35(0.24-0.52)	NA	1	0.37(0.26-0.54)
**Response time**	1-5 week	7	0.35(0.25-0.49)	NA	3	0.41(0.31-0.55)
	6-10 week	6	0.35(0.28-0.45)	NA	3	0.42(0.32-0.55)
	11-18 week	4	0.37(0.24-0.58)	NA	2	0.47(0.30-0.73)
**Country/Region**	China	8	0.40(0.29-0.54)	NA	0	0.40(0.29-0.54)
	China (Taiwan)	6	0.40(0.21-0.74)	NA	2	0.53(0.28-1.04)
	Korea	2	0.52(0.23-1.20)	NA	1	0.36(0.14-0.90)
	Europe	2	0.36(0.20-0.66)	NA	1	0.39(0.23-0.67)
**Patient number**	<100	9	0.39(0.25-0.61)	0.103	3	0.49(0.32-0.74)
	≥100	10	0.41(0.32-0.53)	0.503	0	0.41(0.32-0.53)
**Age(mean/median)**	<60	9	0.39(0.28-0.56)	0.249	2	0.45(0.32-0.64)
	≥60	9	0.45(0.31-0.65)	0.644	1	0.49(0.34-0.70)
**Medication classification**	PD-1 inhibitor	11	0.44(0.32-0.61)	0.733	0	0.44(0.32-0.61)
**Medication**	Atezolizumab	5	0.40(0.28-0.58)	NA	0	0.40(0.28-0.58)
	Nivolumab	2	0.68(0.14-2.86)	NA	1	0.30(0.05-1.68)
**Combination treatment**	At least one combination therapy	7	0.38(0.26-0.54)	NA	0	0.38(0.26-0.54)
	Two or more combination therapy	4	0.51(0.27-0.93)	NA	0	0.51(0.27-0.93)

	TACE/HAIC	4	0.38(0.19-0.78)	NA	0	0.38(0.19-0.78)
	TACE	2	0.57(0.12-2.59)	NA	1	0.26(0.05-1.51)
	HAIC	2	0.28(0.15-0.50)	NA	1	0.34(0.21-0.55)
	TKI	6	0.41(0.27-0.60)	NA	0	0.41(0.27-0.60)
	Lenvatinib	5	0.43(0.26-0.70)	NA	0	0.43(0.26-0.70)
**Effect size**	Univariate	16	0.48(0.36-0.64)	0.410	0	0.48(0.36-0.64)
	Multivariate	12	0.37(0.29-0.47)	0.064	3	0.42(0.33-0.53)
**AFP level**		65	1.60(1.47-1.74)	<0.001	10	1.50(1.37-1.63)
**AFP cutoff**	>400ng/ml	49	1.62(1.46-1.79)	<0.001	8	1.49(1.33-1.67)
	>200ng/ml	9	1.34(0.96-1.87)	NA	0	1.34(0.96-1.87)
	>100ng/ml	4	1.45(1.25-1.68)	NA	1	1.41(1.22-1.62)
	>20ng/ml	3	2.78(1.64-4.71)	NA	0	2.78(1.64-4.71)
	>1000ng/ml	2	1.67(0.71-3.91)	NA	1	2.38(0.95-5.99)
	>500ng/ml	2	1.78(0.80-3.93)	NA	1	1.26(0.53-3.01)
**Medication**	PD-1 inhibitor	38	1.54(1.38-1.72)	0.004	5	1.45(1.29-1.64)
**Medication**	Atezolizumab	14	1.68(1.47-1.91)	0.125	2	1.60(1.39-1.84)
	Camrelizumab	10	1.69(1.24-2.29)	0.087	2	1.48(0.95-2.31)
	Nivolumab	6	1.36(1.13-1.63)	NA	2	1.44(1.21-1.72)
**Combination treatment**	At least one combination therapy	33	1.51(1.33-1.71)	0.189	3	1.48(1.31-1.67)
	Two or more combination therapy	12	1.38(1.16-1.65)	0.411	0	1.38(1.16-1.65)
	TACE/HAIC	12	1.43(1.19-1.71)	0.778	0	1.43(1.19-1.71)
	TACE	8	1.42(1.06-1.91)	NA	0	1.42(1.06-1.91)
	HAIC	3	1.54(0.90-2.65)	NA	0	1.54(0.90-2.65)
	Radiotherapy	3	1.44(0.85-2.42)	NA	0	1.44(0.85-2.42)
	TKI	30	1.49(1.31-1.70)	0.248	2	1.47(1.29-1.66)
	Lenvatinib	14	1.47(1.23-1.76)	0.104	4	1.67(1.39-2.02)
	Apatinib	3	1.88(0.69-5.10)	NA	0	1.88(0.69-5.10)

**Country/Region**	China	36	1.58(1.40-1.78)	0.001	6	1.48(1.32-1.67)
	China (Taiwan)	6	1.53(1.03-2.26)	NA	2	1.92(1.26-2.93)
	Korea	4	1.64(1.23-2.19)	NA	0	1.64(1.23-2.19)
	Japan	7	1.85(1.43-2.37)	NA	2	1.58(1.13-2.20)
	Singapore	2	1.21(0.81-1.80)	NA	1	1.42(0.95-2.13)
	USA	3	1.84(1.09-3.11)	NA	2	1.29(0.74-2.25)
	Multicenter	4	1.56(1.27-1.91)	NA	0	1.56(1.27-1.91)
**Patient number**	<100	25	1.51(1.25-1.81)	0.514	0	1.51(1.25-1.81)
	≥100	40	1.63(1.49-1.79)	<0.001	13	1.47(1.35-1.61)
**Age(mean/median)**	<60	30	1.57(1.36-1.80)	0.003	5	1.47(1.29-1.68)
	≥60	26	1.65(1.47-1.84)	0.033	3	1.57(1.37-1.79)
**Study type**	Retrospective	61	1.60(1.47-1.74)	<0.001	10	1.48(1.36-1.62)
	Prospective	4	1.69(1.13-2.54)	NA	0	1.69(1.13-2.54)
**Effect size**	Univariate	59	1.72(1.56-1.89)	0.163	5	1.62(1.46-1.80)
	Multivariate	31	1.71(1.53-1.90)	<0.001	13	1.49(1.36-1.63)

OS, overall survival; HR, hazard ratios; 95%CI, 95% confidence interval; AFP, alpha-fetoprotein; PD-1, programmed cell death 1; TACE, transhepatic arterial chemotherapy and embolization; HAIC, hepatic artery infusion chemotherapy; TKI, tyrosine kinase inhibitor; NA, not available.

Supplementary Table 5. The trim-and-fill method results of PFS.

Index	Subgroup	Study number	HR (95%CI)	publication bias test P(egger)	Number of additional studies for MetaTrim	Filled HR (95% CI)
**PFS**						
**AFP response**		19	0.38(0.30-0.47)	0.397	0	0.38(0.30-0.47)
**Decrease degree**	>20% decline	12	0.36(0.25-0.52)	0.480	0	0.36(0.25-0.52)
	>50% decline	4	0.36(0.25-0.50)	NA	0	0.36(0.25-0.50)
	>75% decline	3	0.41(0.27-0.61)	NA	2	0.46(0.32-0.65)
	Other	5	0.37(0.29-0.47)	NA	1	0.34(0.26-0.44)
**Response time**	1-5 week	5	0.42(0.27-0.67)	NA	2	0.53(0.33-0.88)
	6-10 week	8	0.32(0.22-0.46)	NA	2	0.28(0.21-0.38)
	11-12 week	2	0.34(0.24-0.48)	NA	1	0.34(0.26-0.46)
**Country/Region**	China	10	0.39(0.27-0.56)	0.822	0	0.39(0.27-0.56)
	China (Taiwan)	4	0.33(0.24-0.45)	NA	0	0.33(0.24-0.45)
	Europe	2	0.40(0.25-0.64)	NA	1	0.41(0.27-0.62)
**Patient number**	<100	10	0.36(0.29-0.45)	0.131	0	0.36(0.29-0.45)
	≥100	9	0.39(0.27-0.57)	NA	0	0.39(0.27-0.57)
**Age(mean/median)**	<60	10	0.44(0.34-0.56)	0.003	5	0.56(0.43-0.73)
	≥60	8	0.39(0.32-0.41)	NA	0	0.39(0.32-0.41)
**Medication**	PD-1 inhibitor	9	0.34(0.25-0.48)	NA	3	0.29(0.22-0.39)
**Medication**	Atezolizumab	6	0.42(0.32-0.55)	NA	2	0.46(0.36-0.59)
**Combination treatment**	At least one combination therapy	8	0.39(0.25-0.60)	NA	0	0.39(0.25-0.60)
	Two or more combination therapy	4	0.47(0.36-0.63)	NA	2	0.54(0.43-0.69)
	TACE/HAIC	5	0.44(0.34-0.57)	NA	2	0.50(0.39-0.63)
	TACE	3	0.48(0.35-0.65)	NA	2	0.58(0.42-0.80)
	HAIC	2	0.37(0.23-0.59)	NA	1	0.31(0.21-0.47)
	TKI	7	0.40(0.24-0.66)	NA	0	0.40(0.24-0.66)

	Lenvatinib	5	0.35(0.19-0.64)	NA	1	0.30(0.18-0.50)
**Study type**	Retrospective	18	0.38(0.31-0.48)	0.515	0	0.38(0.31-0.48)
**Effect size**	Univariate	16	0.39(0.31-0.49)	0.695	0	0.39(0.31-0.49)
	Multivariate	11	0.44(0.35-0.55)	0.004	6	0.57(0.45-0.74)
**AFP level**		54	1.35(1.27-1.42)	0.777	0	1.35(1.27-1.42)
**AFP cutoff**	>400ng/ml	45	1.36(1.27-1.45)	0.669	4	1.33(1.24-1.42)
	>200ng/ml	8	1.37(0.96-1.97)	NA	0	1.37(0.96-1.97)
	>100ng/ml	3	1.44(1.24-1.67)	NA	0	1.44(1.24-1.67)
	>1000ng/ml	2	0.87(0.59-1.29)	NA	1	0.90(0.64-1.28)
**Medication**	PD-1 inhibitor	30	1.37(1.23-1.52)	0.533	4	1.31(1.17-1.46)
**Medication**	Atezolizumab	11	1.39(1.26-1.53)	0.479	2	1.42(1.30-1.56)
	Camrelizumab	9	1.27(0.99-1.62)	NA	0	1.27(0.99-1.62)
	Nivolumab	2	1.21(0.90-1.63)	NA	1	1.10(0.83-1.46)
**Combination treatment**	At least one combination therapy	30	1.30(1.18-1.43)	0.807	2	1.27(1.15-1.40)
	Two or more combination therapy	11	1.50(1.27-1.77)	0.635	1	1.45(1.24-1.70)
	TACE/HAIC	12	1.45(1.20-1.75)	0.914	0	1.45(1.20-1.75)
	TACE	7	1.23(0.94-1.62)	NA	0	1.23(0.94-1.62)
	HAIC	3	1.81(1.27-2.57)	NA	0	1.81(1.27-2.57)
	Radiotherapy	3	1.49(1.00-2.24)	NA	2	1.80(1.29-2.52)
	TKI	26	1.30(1.18-1.44)	0.531	2	1.27(1.14-1.41)
	Lenvatinib	12	1.35(1.17-1.56)	0.998	0	1.35(1.17-1.56)
	Apatinib	2	1.18(0.57-2.44)	NA	1	0.87(0.40-1.92)
**Country/Region**	China	36	1.35(1.24-1.47)	0.838	0	1.35(1.24-1.47)
	China (Taiwan)	4	1.47(1.10-1.98)	NA	0	1.47(1.10-1.98)
	Korea	2	1.34(0.84-2.14)	NA	1	1.10(0.66-1.83)
	Japan	6	1.41(1.24-1.61)	NA	1	1.46(1.29-1.65)
	Multicenter	4	1.30(1.14-1.47)	NA	2	1.25(1.12-1.39)

**Patient number**	<100	21	1.36(1.19-1.55)	0.396	1	1.37(1.19-1.57)
	≥100	33	1.34(1.26-1.44)	0.935	0	1.34(1.26-1.44)
**Age(mean/median)**	<60	29	1.33(1.20-1.46)	0.829	1	1.31(1.19-1.45)
	≥60	17	1.37(1.26-1.48)	0.857	0	1.37(1.26-1.48)
**Study type**	Retrospective	51	1.36(1.28-1.44)	0.934	4	1.33(1.25-1.42)
	Prospective	3	1.10(0.84-1.44)	NA	0	1.10(0.84-1.44)
**Effect size**	Univariate	47	1.35(1.24-1.46)	0.299	0	1.35(1.24-1.46)
	Multivariate	18	1.50(1.39-1.62)	0.956	2	1.48(1.37-1.60)

PFS, progression-free survival; HR, hazard ratios; 95%CI, 95% confidence interval; AFP, alpha-fetoprotein; PD-1, programmed cell death 1; TACE, transhepatic arterial chemotherapy and embolization; HAIC, hepatic artery infusion chemotherapy; TKI, tyrosine kinase inhibitor; NA, not available.

Supplementary Reference

1. Shao, Y.Y., et al., Early alpha-foetoprotein response associated with treatment efficacy of immune checkpoint inhibitors for advanced hepatocellular carcinoma. Liver Int, 2019. 39(11): p. 2184-2189.
2. Chen, S., et al., Association of the Pretreatment Lung Immune Prognostic Index with Survival Outcomes in Advanced Hepatocellular Carcinoma Patients Treated with PD-1 Inhibitors. Journal of Hepatocellular Carcinoma, 2020. Volume 7: p. 289-299.
3. Choi, W.M., et al., Effectiveness and safety of nivolumab in child–pugh b patients with hepatocellular carcinoma: A real-world cohort study. Cancers, 2020. 12(7): p. 1-14.
4. Finn, R.S., et al., Phase ib study of lenvatinib plus pembrolizumab in patients with unresectable hepatocellular carcinoma. Journal of Clinical Oncology, 2020. 38(26): p. 2960-2970.
5. Hsu, W.F., et al., Predictors of response and survival in patients with unresectable hepatocellular carcinoma treated with nivolumab: real-world experience. Am J Cancer Res, 2020. 10(12): p. 4547-4560.
6. Lee, M.S., et al., Atezolizumab with or without bevacizumab in unresectable hepatocellular carcinoma (GO30140): an open-label, multicentre, phase 1b study. Lancet Oncol, 2020. 21(6): p. 808-820.
7. Lee, P.C., et al., Predictors of Response and Survival in Immune Checkpoint Inhibitor-Treated Unresectable Hepatocellular Carcinoma. Cancers (Basel), 2020. 12(1).
8. Spahn, S., et al., Clinical and Genetic Tumor Characteristics of Responding and Non-Responding Patients to PD-1 Inhibition in Hepatocellular Carcinoma. Cancers (Basel), 2020. 12(12).
9. Sung, P.S., et al., Real-World Outcomes of Nivolumab in Patients With Unresectable Hepatocellular Carcinoma in an Endemic Area of Hepatitis B Virus Infection. Frontiers in Oncology, 2020. 10.
10. Yuan, G., et al., Safety and Efficacy of Camrelizumab Combined with Apatinib for Advanced Hepatocellular Carcinoma with Portal Vein Tumor Thrombus: A Multicenter Retrospective Study. Onco Targets Ther, 2020. 13: p. 12683-12693.
11. Choi, W.M., et al., Kinetics of the neutrophil-lymphocyte ratio during PD-1 inhibition as a prognostic factor in advanced hepatocellular carcinoma. Liver International, 2021. 41(9): p. 2189-2199.
12. Hsu, W.F., et al., Alpha-fetoprotein response predicts treatment outcomes in patients with unresectable hepatocellular carcinoma receiving immune checkpoint inhibitors with or without tyrosine kinase inhibitors or locoregional therapies. Am J Cancer Res, 2021. 11(12): p. 6173-6187.
13. Mei, J., et al., Hepatic Arterial Infusion Chemotherapy Combined With PD-1 Inhibitors Plus Lenvatinib Versus PD-1 Inhibitors Plus Lenvatinib for Advanced Hepatocellular Carcinoma. Frontiers in Oncology, 2021. 11.
14. Morita, M., et al., Immunological Microenvironment Predicts the Survival of the Patients with Hepatocellular Carcinoma Treated with Anti-PD-1 Antibody. Liver Cancer, 2021. 10(4): p. 380-393.

15. Ng, K.Y.Y., et al., Real-world efficacy and safety of immune checkpoint inhibitors in advanced hepatocellular carcinoma: Experience of a tertiary Asian Center. Asia Pac J Clin Oncol, 2021. 17(5): p. e249-e261.
16. Pinato, D.J., et al., Treatment-related toxicity and improved outcome from immunotherapy in hepatocellular cancer: Evidence from an FDA pooled analysis of landmark clinical trials with validation from routine practice. Eur J Cancer, 2021. 157: p. 140-152.
17. Sun, X., et al., Reductions in AFP and PIVKA-II can predict the efficiency of anti-PD-1 immunotherapy in HCC patients. BMC Cancer, 2021. 21(1): p. 775.
18. Teng, W., et al., Alpha-fetoprotein response at different time-points is associated with efficacy of nivolumab monotherapy for unresectable hepatocellular carcinoma. Am J Cancer Res, 2021. 11(5): p. 2319-2330.
19. Chen, J., et al., Body mass index, as a novel predictor of hepatocellular carcinoma patients treated with Anti-PD-1 immunotherapy. Frontiers in Medicine, 2022. 9.
20. Chen, S.C., et al., Anti-PD-1 combined sorafenib versus anti-PD-1 alone in the treatment of advanced hepatocellular cell carcinoma: a propensity score-matching study. BMC Cancer, 2022. 22(1): p. 55.
21. Cheon, J., et al., Efficacy and safety of atezolizumab plus bevacizumab in Korean patients with advanced hepatocellular carcinoma. Liver Int, 2022. 42(3): p. 674-681.
22. Chuma, M., et al., Safety and efficacy of atezolizumab plus bevacizumab in patients with unresectable hepatocellular carcinoma in early clinical practice: A multicenter analysis. Hepatology Research, 2022. 52(3): p. 269-280.
23. Dong, D., et al., Prognostic significance of albumin-bilirubin score in patients with unresectable hepatocellular carcinoma undergoing combined immunotherapy and radiotherapy. J Med Imaging Radiat Oncol, 2022. 66(5): p. 662-670.
24. Guo, Z., et al., The efficacy and safety of conventional transcatheter arterial chemoembolization combined with PD-1 inhibitor and anti-angiogenesis tyrosine kinase inhibitor treatment for patients with unresectable hepatocellular carcinoma: a real-world comparative study. Frontiers in Oncology, 2022. 12.
25. Hayakawa, Y., et al., Early experience of atezolizumab plus bevacizumab therapy in Japanese patients with unresectable hepatocellular carcinoma in real-world practice. Invest New Drugs, 2022. 40(2): p. 392-402.
26. Huang, J.T., et al., Transarterial Chemoembolization Combined with Immune Checkpoint Inhibitors Plus Tyrosine Kinase Inhibitors versus Immune Checkpoint Inhibitors Plus Tyrosine Kinase Inhibitors for Advanced Hepatocellular Carcinoma. Journal of Hepatocellular Carcinoma, 2022. 9: p. 1217-1228.
27. Huang, R., et al., Blood Biomarkers Predict Survival Outcomes in Patients with Hepatitis B Virus-Induced Hepatocellular Carcinoma Treated with PD-1 Inhibitors. J Immunol Res, 2022. 2022: p. 3781109.

28. Ju, S., et al., Late combination of transarterial chemoembolization with apatinib and camrelizumab for unresectable hepatocellular carcinoma is superior to early combination. BMC Cancer, 2022. 22(1).
29. Ju, S., et al., Apatinib Plus Camrelizumab With/Without Chemoembolization for Hepatocellular Carcinoma: A Real-World Experience of a Single Center. Frontiers in Oncology, 2022. 11.
30. Kim, H.I., J. Lim, and J.H. Shim, Role of the alpha-fetoprotein response in immune checkpoint inhibitor-based treatment of patients with hepatocellular carcinoma. J Cancer Res Clin Oncol, 2022. 148(8): p. 2069-2077.
31. Kim, H.S., et al., The presence and size of intrahepatic tumors determine the therapeutic efficacy of nivolumab in advanced hepatocellular carcinoma. Ther Adv Med Oncol, 2022. 14: p. 17588359221113266.
32. Lee, S.W., et al., The Combining of Tyrosine Kinase Inhibitors and Immune Checkpoint Inhibitors as First-Line Treatment for Advanced Stage Hepatocellular Carcinoma. Journal of Clinical Medicine, 2022. 11(16).
33. Lewis, S., et al., Comparative assessment of standard and immune response criteria for evaluation of response to PD-1 monotherapy in unresectable HCC. Abdom Radiol (NY), 2022. 47(3): p. 969-980.
34. Li, X., et al., Efficacy and Safety of Lenvatinib Combined With PD-1 Inhibitors Plus TACE for Unresectable Hepatocellular Carcinoma Patients in China Real-World. Frontiers in Oncology, 2022. 12.
35. Liu, H., et al., Comparison of effectiveness and safety of camrelizumab between HBV-related and non-B, non-C hepatocellular carcinoma: A retrospective study in China. Frontiers in Genetics, 2022. 13.
36. Matsumoto, H., et al., Clinical Usefulness of Monitoring Muscle Volume during Atezolizumab Plus Bevacizumab Therapy in Patients with Unresectable Hepatocellular Carcinoma. Cancers, 2022. 14(14).
37. Peng, T.R., et al., Therapeutic efficacy of nivolumab plus sorafenib therapy in patients with unresectable hepatocellular carcinoma. Int Immunopharmacol, 2022. 112: p. 109223.
38. Su, C.W., et al., Proton beam radiotherapy combined with anti-PD1/PDL1 immune checkpoint inhibitors for advanced hepatocellular carcinoma. Am J Cancer Res, 2022. 12(4): p. 1606-1620.
39. Sun, X., et al., Real-world efficiency of lenvatinib plus PD-1 blockades in advanced hepatocellular carcinoma: an exploration for expanded indications. BMC Cancer, 2022. 22(1).
40. Tamaki, N., et al., Optimal threshold of alpha-fetoprotein response in patients with unresectable hepatocellular carcinoma treated with atezolizumab and bevacizumab. Invest New Drugs, 2022. 40(6): p. 1290-1297.
41. Teng, W., et al., Combination of CRAFITY score with Alpha-fetoprotein response predicts a favorable outcome of atezolizumab plus bevacizumab for unresectable hepatocellular carcinoma.

Am J Cancer Res, 2022. 12(4): p. 1899-1911.
42. Wu, Y.L., et al., Neutrophil-to-Lymphocyte and Platelet-to-Lymphocyte Ratios as Prognostic Biomarkers in Unresectable Hepatocellular Carcinoma Treated with Atezolizumab plus Bevacizumab. Cancers, 2022. 14(23).
43. Xiang, Y.J., et al., Transarterial chemoembolization plus a PD-1 inhibitor with or without lenvatinib for intermediate-stage hepatocellular carcinoma. Hepatology Research, 2022. 52(8): p. 721-729.
44. Yao, J., et al., Efficacy and safety of PD-1 inhibitor combined with antiangiogenic therapy for unresectable hepatocellular carcinoma: A multicenter retrospective study. Cancer Med, 2022. 11(19): p. 3612-3622.
45. You, R., et al., Efficacy and safety of camrelizumab plus transarterial chemoembolization in intermediate to advanced hepatocellular carcinoma patients: A prospective, multi-center, real-world study. Frontiers in Oncology, 2022. 12.
46. Zhang, Z., et al., Prognostic value of inflammation-immunity-nutrition score in patients with hepatocellular carcinoma treated with anti-PD-1 therapy. Journal of Clinical Laboratory Analysis, 2022. 36(5).
47. Zhao, M., et al., Sarcopenia and Systemic Inflammation Response Index Predict Response to Systemic Therapy for Hepatocellular Carcinoma and Are Associated With Immune Cells. Frontiers in Oncology, 2022. 12.
48. Zhu, A.X., et al., Alpha-Fetoprotein as a Potential Surrogate Biomarker for Atezolizumab plus Bevacizumab Treatment of Hepatocellular Carcinoma. CLINICAL CANCER RESEARCH, 2022. 28(16): p. 3537-3545.
49. Akyildiz, A., et al., The safety and efficacy of first-line atezolizumab plus bevacizumab in patients with unresectable hepatocellular carcinoma: A multicenter real-world study from Turkey. Medicine (Baltimore), 2023. 102(45): p. e35950.
50. Campani, C., et al., Baseline ALBI score and early variation of serum AFP predicts outcomes in patients with HCC treated by atezolizumab–bevacizumab. Liver International, 2023. 43(3): p. 708-717.
51. Cheon, J., et al., Atezolizumab plus bevacizumab in patients with child–Pugh B advanced hepatocellular carcinoma. Therapeutic Advances in Medical Oncology, 2023. 15.
52. Cheung, C.C.L., et al., Immunohistochemical scoring of LAG-3 in conjunction with CD8 in the tumor microenvironment predicts response to immunotherapy in hepatocellular carcinoma. Frontiers in Immunology, 2023. 14.
53. Chiang, H.C., et al., Real-World Effectiveness of Sorafenib versus Lenvatinib Combined with PD-1 Inhibitors in Unresectable Hepatocellular Carcinoma. Cancers, 2023. 15(3).
54. Fukushima, T., et al., Association Between Immune-Related Adverse Events and Survival in Patients with Hepatocellular Carcinoma Treated With Atezolizumab Plus Bevacizumab. Oncologist, 2023. 28(7): p. E526-E533.

55. Hong, C., et al., Predictive Value of the Hepatic Immune Predictive Index for Patients with Primary Liver Cancer Treated with Immune Checkpoint Inhibitors. Dig Dis, 2023. 41(3): p. 422-430.
56. Hsu, W.F., et al., Combined CRAFITY score and α-fetoprotein response predicts treatment outcomes in patients with unresectable hepatocellular carcinoma receiving anti-programmed death-1 blockade-based immunotherapy. Am J Cancer Res, 2023. 13(2): p. 654-668.
57. Jia, G., et al., Nomogram for predicting survival in patients with advanced hepatocellular carcinoma treated with PD-1 inhibitors: incorporating pre-treatment and post-treatment clinical parameters. BMC Cancer, 2023. 23(1).
58. Kang, S., et al., Impact of metformin on clinical outcomes in advanced hepatocellular carcinoma treated with immune checkpoint inhibitors. Liver Cancer International, 2023. 4(2): p. 77-88.
59. Li, G., et al., Effectiveness and Safety of the PD-1 Inhibitor Lenvatinib Plus Radiotherapy in Patients with HCC with Main PVTT: Real-World Data from a Tertiary Centre. J Hepatocell Carcinoma, 2023. 10: p. 2037-2048.
60. Li, H., et al., PD-1 Inhibitors Combined with Antiangiogenic Therapy with or Without Transarterial Chemoembolization in the Treatment of Hepatocellular Carcinoma: A Propensity Matching Analysis. J Hepatocell Carcinoma, 2023. 10: p. 1257-1266.
61. Li, J., et al., Simultaneous and Sequential Use of Molecular Targeted Agents Plus Immune Checkpoint Inhibitors for Advanced Hepatocellular Carcinoma: A Real-World Practice in China. Journal of Hepatocellular Carcinoma, 2023. 10: p. 949-958.
62. Li, Q., F. Ma, and J.f. Wang, Advanced lung cancer inflammation index predicts survival outcomes of hepatocellular carcinoma patients receiving immunotherapy. FRONTIERS IN ONCOLOGY, 2023. 13.
63. Li, S., et al., Prediction of early treatment response to the combination therapy of TACE plus lenvatinib and anti-PD-1 antibody immunotherapy for unresectable hepatocellular carcinoma: Multicenter retrospective study. FRONTIERS IN IMMUNOLOGY, 2023. 14.
64. Liu, C., et al., Prognostic value of nutritional and inflammatory markers in patients with hepatocellular carcinoma who receive immune checkpoint inhibitors. Oncology Letters, 2023. 26(4).
65. Long, T., et al., Comparable Clinical Outcomes Between Transarterial Chemoembolization or Hepatic Arterial Infusion Chemotherapy Combined with Tyrosine Kinase Inhibitors and PD-1 Inhibitors in Unresectable Hepatocellular Carcinoma. Journal of Hepatocellular Carcinoma, 2023. 10: p. 1849-1859.
66. Lu, L., et al., Trajectories of α-fetoprotein and unresectable hepatocellular carcinoma outcomes receiving atezolizumab plus bevacizumab: a secondary analysis of IMbrave150 study. Br J Cancer, 2023. 129(4): p. 620-625.
67. Luo, M.C., et al., Early Tumor Marker Response Predicts Treatment Outcomes in Patients with Unresectable Hepatocellular Carcinoma Receiving Combined Lenvatinib, Immune Checkpoint

Inhibitors, and Transcatheter Arterial Chemoembolization Therapy. J Hepatocell Carcinoma, 2023. 10: p. 1827-1837.
68. Navadurong, H., et al., Modified albumin-bilirubin predicted survival of unresectable hepatocellular carcinoma patients treated with immunotherapy. World Journal of Gastrointestinal Oncology, 2023. 15(10): p. 1771-1783.
69. Pan, Y., et al., Factors influencing the prognosis patients with Barcelona Clinic Liver Cancer stage C hepatocellular carcinoma undergoing salvage surgery after conversion therapy. Transl Cancer Res, 2023. 12(7): p. 1852-1862.
70. Persano, M., et al., Clinical outcomes with atezolizumab plus bevacizumab or lenvatinib in patients with hepatocellular carcinoma: a multicenter real-world study. Journal of Cancer Research and Clinical Oncology, 2023. 149(9): p. 5591-5602.
71. Qu, S., D. Wu, and Z. Hu, Neutrophil-to-Lymphocyte Ratio and Early Tumor Shrinkage as Predictive Biomarkers in Unresectable Hepatocellular Carcinoma Patients Treated With Lenvatinib, PD-1 Inhibitors, in Combination With TACE. Technology in Cancer Research and Treatment, 2023. 22.
72. Raj, R., et al., Immunotherapy for Advanced Hepatocellular Carcinoma-a Large Tertiary Center Experience. J Gastrointest Surg, 2023. 27(10): p. 2126-2134.
73. Sun, T., et al., Association of the pretreatment lung immune prognostic index with immune checkpoint inhibitor outcomes in patients with advanced hepatocellular carcinoma. European Journal of Medical Research, 2023. 28(1).
74. Tanabe, N., et al., Early Prediction of Response Focused on Tumor Markers in Atezolizumab plus Bevacizumab Therapy for Hepatocellular Carcinoma. Cancers (Basel), 2023. 15(11).
75. Tang, C., et al., Portal vein tumour thrombosis radiotherapy improves the treatment outcomes of immunotherapy plus bevacizumab in hepatocellular carcinoma: a multicentre real-world analysis with propensity score matching. Frontiers in Immunology, 2023. 14.
76. Wang, J., et al., Transarterial Chemoembolization Combined With PD-1 Inhibitors Plus Lenvatinib Showed Improved Efficacy for Treatment of Unresectable Hepatocellular Carcinoma Compared With PD-1 Inhibitors Plus Lenvatinib. Technology in Cancer Research and Treatment, 2023. 22.
77. Wu, Y.L., et al., Outcomes of beta blocker use in advanced hepatocellular carcinoma treated with immune checkpoint inhibitors. Frontiers in Oncology, 2023. 13.
78. Xiao, Y., et al., Pretreatment Neutrophil-to-Lymphocyte Ratio as Prognostic Biomarkers in Patients with Unresectable Hepatocellular Carcinoma Treated with Hepatic Arterial Infusion Chemotherapy Combined with Lenvatinib and Camrelizumab. J Hepatocell Carcinoma, 2023. 10: p. 2049-2058.
79. Xin, H., et al., Noninvasive evaluation of neutrophil extracellular traps signature predicts clinical outcomes and immunotherapy response in hepatocellular carcinoma. Frontiers in Immunology, 2023. 14.

80. Xu, L., et al., Alkaline phosphatase combined with γ-glutamyl transferase is an independent predictor of prognosis of hepatocellular carcinoma patients receiving programmed death-1 inhibitors. Frontiers in Immunology, 2023. 14.
81. Xu, M.H., et al., Effectiveness and safety of lenvatinib plus anti-programmed death-1 antibodies in patients with hepatocellular carcinoma: A real-world cohort study. Cancer Medicine, 2023. 12(8): p. 9202-9212.
82. Yang, X., et al., Efficacy and Safety of Regorafenib Plus Immune Checkpoint Inhibitors with or Without TACE as a Second-Line Treatment for Advanced Hepatocellular Carcinoma: A Propensity Score Matching Analysis. J Hepatocell Carcinoma, 2023. 10: p. 303-313.
83. Yano, Y., et al., Factors associated with the response to atezolizumab/bevacizumab combination therapy for hepatocellular carcinoma. JGH Open, 2023. 7(7): p. 476-481.
84. Yin, Y., et al., Treatment with camrelizumab plus tyrosine kinase inhibitors with or without TACE for intermediate-advanced hepatocellular carcinoma: A clinical efficacy and safety study. Oncologie, 2023. 25(3): p. 257-267.
85. Yu, B., et al., Tyrosine Kinase Inhibitors Plus Anti-PD-1 Antibodies with Hepatic Arterial Infusion Chemotherapy or Transarterial Chemoembolization for Unresectable Hepatocellular Carcinoma. J Hepatocell Carcinoma, 2023. 10: p. 1735-1748.
86. Zhang, W., et al., Lenvatinib plus anti-PD-1 antibodies as conversion therapy for patients with unresectable intermediate-advanced hepatocellular carcinoma: A single-arm, phase II trial. Journal for ImmunoTherapy of Cancer, 2023. 11(9).
87. Zhang, W., et al., Hepatic arterial infusion chemotherapy combined with anti-PD-1/PD-L1 immunotherapy and molecularly targeted agents for advanced hepatocellular carcinoma: a real world study. FRONTIERS IN IMMUNOLOGY, 2023. 14.
88. Zhang, Y., et al., Development and Assessment of Nomogram Based on AFP Response for Patients with Unresectable Hepatocellular Carcinoma Treated with Immune Checkpoint Inhibitors. Cancers (Basel), 2023. 15(21).
89. Zhu, H.-F., et al., Combination of alpha-fetoprotein and neutrophil-to-lymphocyte ratio to predict treatment response and survival outcomes of patients with unresectable hepatocellular carcinoma treated with immune checkpoint inhibitors. BMC CANCER, 2023. 23(1).
90. Cai, H., et al., Atezolizumab Plus Bevacizumab Combined with Transarterial Embolization Plus Hepatic Arterial Infusion Chemotherapy for Unresectable Hepatocellular Carcinoma with a Diameter >8 Cm: A Retrospective Study. Journal of Hepatocellular Carcinoma, 2024. 11: p. 399-409.
91. Chen, B.B., et al., Changes in Posttreatment Spleen Volume Associated with Immunotherapy Outcomes for Advanced Hepatocellular Carcinoma. Journal of Hepatocellular Carcinoma, 2024. 11: p. 1015-1029.
92. Chen, J.L., et al., Prognostic value of circulating tumor cells combined with neutrophil-lymphocyte ratio in patients with hepatocellular carcinoma. World J Gastrointest Oncol, 2024. 16(2):

p. 372-385.
93. Chen, Y., et al., Taurohyocholic acid acts as a potential predictor of the efficacy of tyrosine kinase inhibitors combined with programmed cell death-1 inhibitors in hepatocellular carcinoma. Frontiers in Pharmacology, 2024. 15.
94. Chuma, M., et al., Clinical significance of circulating biomarkers of immune-checkpoint molecules with atezolizumab plus bevacizumab therapy in unresectable hepatocellular carcinoma. Hepatology International, 2024. 18(5): p. 1472-1485.
95. Copil, F.D., et al., No correlation between MASLD and poor outcome of Atezolizumab-Bevacizumab therapy in patients with advanced HCC. Liver Int, 2024. 44(4): p. 931-943.
96. Diao, L., et al., Hepatic arterial infusion chemotherapy combined with lenvatinib and PD-1 inhibitors versus lenvatinib and PD-1 inhibitors for HCC refractory to TACE. Journal of Gastroenterology and Hepatology (Australia), 2024. 39(4): p. 746-753.
97. Fu, S., et al., Hepatic arterial infusion chemotherapy, lenvatinib plus programmed cell death protein-1 inhibitors: A promising treatment approach for high-burden hepatocellular carcinoma. Cancer Medicine, 2024. 13(9).
98. Guo, Y., et al., Prognostic value of platelet-to-lymphocyte ratio in patients with unresectable hepatocellular carcinoma undergoing transarterial chemoembolization and tyrosine kinase inhibitors plus immune checkpoints inhibitors. FRONTIERS IN ONCOLOGY, 2024. 14.
99. Han, J., et al., Impact of metabolic dysfunction-associated steatotic liver disease on the efficacy of immunotherapy in patients with chronic hepatitis B-related hepatocellular carcinoma. Cancer Biol Med, 2024.
100. He, M., et al., A Novel Nomogram to Predict Prognosis of Advanced Hepatocellular Carcinoma Treated with Intensity-Modulated Radiotherapy Plus Anti-PD1. J Hepatocell Carcinoma, 2024. 11: p. 913-925.
101. Huang, Z., et al., The safety and efficacy of TACE combined with HAIC, PD-1 inhibitors, and tyrosine kinase inhibitors for unresectable hepatocellular carcinoma: a retrospective study. Frontiers in Oncology, 2024. 14.
102. Kai, M., et al., Clinical factors associated with the therapeutic efficacy of atezolizumab plus bevacizumab in patients with unresectable hepatocellular carcinoma: A multicenter prospective observational study. PLoS ONE, 2024. 19(1 January).
103. Kaneko, S., et al., Prognostic significance of C-reactive protein in unresectable hepatocellular carcinoma treated with atezolizumab and bevacizumab. Hepatol Res, 2024. 54(6): p. 562-574.
104. Kuzuya, T., et al., Early Changes in Alpha-Fetoprotein and Des-gamma-Carboxy Prothrombin Are Useful Predictors of Antitumor Response to Durvalumab Plus Tremelimumab Therapy for Advanced Hepatocellular Carcinoma. Current oncology (Toronto, Ont.), 2024. 31(8): p. 4225-4240.
105. Lee, S.W., S.S. Yang, and T.Y. Lee, A Real-World Experience on a Chinese Population of Patients With Unresectable Hepatocellular Carcinoma Treated With Nivolumab. Gastroenterology Res, 2024. 17(1): p. 15-22.

106. Li, J., et al., Real-world study of hepatic artery infusion chemotherapy combined with anti-PD-1 immunotherapy for hepatocellular carcinoma patients with portal vein tumor thrombus. Therapeutic Advances in Medical Oncology, 2024. 16.
107. Li, R., et al., Hepatic Arterial Infusion Chemotherapy Combined Lenvatinib and PD-1 Inhibitor Showed Improved Survival for Infiltrative Hepatocellular Carcinoma: A Multicenter Cohort Study. J Hepatocell Carcinoma, 2024. 11: p. 1727-1740.
108. Li, Y., et al., Hepatic artery infusion chemotherapy combined with camrelizumab plus rivoceranib for hepatocellular carcinoma with portal vein tumor thrombosis: a multicenter propensity score-matching analysis. Hepatol Int, 2024. 18(4): p. 1286-1298.
109. Lin, K.-Y., et al., Serum alpha-fetoprotein response as a preoperative prognostic indicator in unresectable hepatocellular carcinoma with salvage hepatectomy following conversion therapy: a multicenter retrospective study. FRONTIERS IN IMMUNOLOGY, 2024. 15.
110. Liu, J., et al., Real-world status, efficacy and prognosis analysis of first-line treatment for unresectable hepatocellular carcinoma in patients treated at multiple centres. Annals of Medicine, 2024. 56(1).
111. Lu, Y. and Y. Lu, Clinical predictive factors of the efficacy of immune checkpoint inhibitors and kinase inhibitors in advanced hepatocellular cancer. Clin Transl Oncol, 2024.
112. Ma, K.P., et al., Efficacy and predictive factors of transarterial chemoembolization combined with lenvatinib plus programmed cell death protein-1 inhibition for unresectable hepatocellular carcinoma. World J Gastrointest Oncol, 2024. 16(4): p. 1236-1247.
113. Ma, W., et al., Blood MALT1 serves as a potential biomarker reflecting the response and survival of immune-checkpoint-inhibitor therapy in advanced hepatocellular carcinoma. ONCOLOGY LETTERS, 2024. 28(4).
114. Mo, Z., et al., Prognostic model for unresectable hepatocellular carcinoma treated with dual PD-1 and angiogenesis blockade therapy. J Immunother Cancer, 2024. 12(1).
115. Moriyama, E., et al., Efficacy of Atezolizumab Plus Bevacizumab-Transcatheter Arterial Chemoembolization Sequential Therapy for Patients with Intermediate-Stage Hepatocellular Carcinoma. Curr Oncol, 2024. 31(10): p. 5821-5831.
116. Nakabori, T., et al., Safety and Feasibility of Combining On-Demand Selective Locoregional Treatment with First-Line Atezolizumab Plus Bevacizumab for Patients with Unresectable Hepatocellular Carcinoma. Current Oncology, 2024. 31(3): p. 1543-1555.
117. Nakazawa, M., et al., Impact of Neoadjuvant Immunotherapy on Recurrence-Free Survival in Patients with High-Risk Localized HCC. CANCER RESEARCH COMMUNICATIONS, 2024. 4(8): p. 2123-2132.
118. Rossari, F., et al., Disease Etiology Impact on Outcomes of Hepatocellular Carcinoma Patients Treated with Atezolizumab plus Bevacizumab: A Real-World, Multicenter Study. Liver Cancer, 2024.
119. Saeki, I., et al., Alpha-fetoprotein and des-gamma-carboxy prothrombin can predict the

objective response of patients with hepatocellular carcinoma receiving durvalumab plus tremelimumab therapy. PLoS ONE, 2024. 19(9).
120. Sobirey, R., et al., Tumor response assessment in hepatocellular carcinoma treated with immunotherapy: imaging biomarkers for clinical decision-making. Eur Radiol, 2024.
121. Sun, W., et al., The clinical significance of sarcopenia in patients with hepatocellular carcinoma treated with lenvatinib and PD-1 inhibitors. Frontiers in Immunology, 2024. 15.
122. Tada, T., et al., Outcomes of patients with hepatocellular carcinoma treated with atezolizumab plus bevacizumab in real-world clinical practice who met or did not meet the inclusion criteria for the phase 3 IMbrave150 trial. Aliment Pharmacol Ther, 2024. 60(2): p. 233-245.
123. Wang, L., et al., Salvage Surgery for Initially Unresectable HCC With PVTT Converted by Locoregional Treatment Plus Tyrosine Kinase Inhibitor and Anti-PD-1 Antibody. Oncologist, 2024. 29(8): p. e1041-e1050.
124. Wang, Y.Q., et al., Impact of baseline body mass index on the long-term prognosis of advanced hepatocellular carcinoma treated with immunotherapy. World J Gastroenterol, 2024. 30(37): p. 4132-4148.
125. Xiao, Y., et al., Prognostic Value of Alpha-Fetoprotein in Unresectable Hepatocellular Carcinoma Treated with Hepatic Artery Infusion Chemotherapy Combined with Lenvatinib and Camrelizumab. J Hepatocell Carcinoma, 2024. 11: p. 1251-1263.
126. Xin, Y., et al., Gut microbiota as a prognostic biomarker for unresectable hepatocellular carcinoma treated with anti-PD-1 therapy. Frontiers in Genetics, 2024. 15.
127. Xu, L., et al., Efficacy and safety of tislelizumab plus lenvatinib as first-line treatment in patients with unresectable hepatocellular carcinoma: a multicenter, single-arm, phase 2 trial. BMC Medicine, 2024. 22(1).
128. Yang, J., et al., Dynamic Changes of Neutrophil-to-Lymphocyte Ratio on Predicting Response of Immune Checkpoint Inhibitors Plus Targeted Therapies for Unresectable Hepatocellular Carcinoma. J Hepatocell Carcinoma, 2024. 11: p. 1495-1505.
129. Yao, Y., et al., Changes in systemic immune-inflammation index (SII) predict the prognosis of patients with hepatitis B-related hepatocellular carcinoma treated with lenvatinib plus PD-1 inhibitors. Clin Transl Oncol, 2024.
130. Zheng, X., et al., Evaluating the impact of treatment sequencing on outcomes in hepatocellular carcinoma: a comparative analysis of TACE and systemic therapies. Clinical and Experimental Medicine, 2024. 24(1).
131. Zuo, M., et al., Hepatic arterial infusion chemotherapy plus camrelizumab and apatinib for advanced hepatocellular carcinoma. Hepatology International, 2024. 18(5): p. 1486-1498.

PRISMA 2020 Checklist

Section and Topic	Item #	Checklist item	Location where item is reported
**TITLE**			
Title	1	Identify the report as a systematic review.	Page 1
**ABSTRACT**			
Abstract	2	See the PRISMA 2020 for Abstracts checklist.	Page 2
**INTRODUCTION**			
Rationale	3	Describe the rationale for the review in the context of existing knowledge.	Page 3-4
Objectives	4	Provide an explicit statement of the objective(s) or question(s) the review addresses.	Page 3-4
**METHODS**			
Eligibility criteria	5	Specify the inclusion and exclusion criteria for the review and how studies were grouped for the syntheses.	Page 5-6
Information sources	6	Specify all databases, registers, websites, organisations, reference lists and other sources searched or consulted to identify studies. Specify the date when each source was last searched or consulted.	Page 5
Search strategy	7	Present the full search strategies for all databases, registers and websites, including any filters and limits used.	Page 5
Selection process	8	Specify the methods used to decide whether a study met the inclusion criteria of the review, including how many reviewers screened each record and each report retrieved, whether they worked independently, and if applicable, details of automation tools used in the process.	Page 5 Figure1
Data collection process	9	Specify the methods used to collect data from reports, including how many reviewers collected data from each report, whether they worked independently, any processes for obtaining or confirming data from study investigators, and if applicable, details of automation tools used in the process.	Page 6
Data items	10a	List and define all outcomes for which data were sought. Specify whether all results that were compatible with each outcome domain in each study were sought (e.g. for all measures, time points, analyses), and if not, the methods used to decide which results to collect.	Page 6-8
	10b	List and define all other variables for which data were sought (e.g. participant and intervention characteristics, funding sources). Describe any assumptions made about any missing or unclear information.	Page 6-8
Study risk of bias assessment	11	Specify the methods used to assess risk of bias in the included studies, including details of the tool(s) used, how many reviewers assessed each study and whether they worked independently, and if applicable, details of automation tools used in the process.	Page 6-8
Effect measures	12	Specify for each outcome the effect measure(s) (e.g. risk ratio, mean difference) used in the synthesis or presentation of results.	Page 6-8
Synthesis methods	13a	Describe the processes used to decide which studies were eligible for each synthesis (e.g. tabulating the study intervention characteristics and comparing against the planned groups for each synthesis (item #5)).	Page 6-8
	13b	Describe any methods required to prepare the data for presentation or synthesis, such as handling of missing summary statistics, or data conversions.	Page 6-8
	13c	Describe any methods used to tabulate or visually display results of individual studies and syntheses.	Page 6-8
	13d	Describe any methods used to synthesize results and provide a rationale for the choice(s). If meta-analysis was performed, describe the model(s), method(s) to identify the presence and extent of statistical heterogeneity, and software package(s) used.	Page 6-8
	13e	Describe any methods used to explore possible causes of heterogeneity among study results (e.g. subgroup analysis, meta-regression).	Page 6-8
	13f	Describe any sensitivity analyses conducted to assess robustness of the synthesized results.	Page 6-8
Reporting bias assessment	14	Describe any methods used to assess risk of bias due to missing results in a synthesis (arising from reporting biases).	Page 6-8
Certainty assessment	15	Describe any methods used to assess certainty (or confidence) in the body of evidence for an outcome.	Page 6-8

PRISMA 2020 Checklist

Section and Topic	Item #	Checklist item	Location where item is reported
**RESULTS**			
Study selection	16a	Describe the results of the search and selection process, from the number of records identified in the search to the number of studies included in the review, ideally using a flow diagram.	Page 8-9
	16b	Cite studies that might appear to meet the inclusion criteria, but which were excluded, and explain why they were excluded.	Page 8-9
Study characteristics	17	Cite each included study and present its characteristics.	Page 8-9
Risk of bias in studies	18	Present assessments of risk of bias for each included study.	Page 9-15
Results of individual studies	19	For all outcomes, present, for each study: (a) summary statistics for each group (where appropriate) and (b) an effect estimate and its precision (e.g. confidence/credible interval), ideally using structured tables or plots.	Page 9-15
Results of syntheses	20a	For each synthesis, briefly summarise the characteristics and risk of bias among contributing studies.	Page 9-15
	20b	Present results of all statistical syntheses conducted. If meta-analysis was done, present for each the summary estimate and its precision (e.g. confidence/credible interval) and measures of statistical heterogeneity. If comparing groups, describe the direction of the effect.	Page 9-15
	20c	Present results of all investigations of possible causes of heterogeneity among study results.	Page 9-15
	20d	Present results of all sensitivity analyses conducted to assess the robustness of the synthesized results.	Page 9-15
Reporting biases	21	Present assessments of risk of bias due to missing results (arising from reporting biases) for each synthesis assessed.	Page 9-15
Certainty of evidence	22	Present assessments of certainty (or confidence) in the body of evidence for each outcome assessed.	Page 9-15
**DISCUSSION**			
Discussion	23a	Provide a general interpretation of the results in the context of other evidence.	Page 15-18
	23b	Discuss any limitations of the evidence included in the review.	Page 15-18
	23c	Discuss any limitations of the review processes used.	Page 15-18
	23d	Discuss implications of the results for practice, policy, and future research.	Page 15-18
**OTHER INFORMATION**			
Registration and protocol	24a	Provide registration information for the review, including register name and registration number, or state that the review was not registered.	CRD-42024606729
	24b	Indicate where the review protocol can be accessed, or state that a protocol was not prepared.	Protocol prepared
	24c	Describe and explain any amendments to information provided at registration or in the protocol.	Page 18-19
Support	25	Describe sources of financial or non-financial support for the review, and the role of the funders or sponsors in the review.	Page 18-19
Competing interests	26	Declare any competing interests of review authors.	Page 18-19
Availability of data, code and other materials	27	Report which of the following are publicly available and where they can be found: template data collection forms; data extracted from included studies; data used for all analyses; analytic code; any other materials used in the review.	All materials are publicly available
