# Supplementary material for: The prognostic and predictive value of AFP in immune checkpoint inhibitor-treated hepatocellular carcinoma: a systematic review and meta-analysis
Source: Front Immunol. 2025 Nov 4;16:1695861. doi: 10.3389/fimmu.2025.1695861 (PMC12623344; doi:10.3389/fimmu.2025.1695861)
Supplement: Supplementary file 2 [file DataSheet2.pdf]

Supplementary Table. Database-specific search strategy

P (population)	Keywords searched for in abstract and title	"Carcinoma, Hepatocellular" OR "hepatocellular carcinoma*" OR "hepatocellular cancer" OR "hepatic carcinoma*" OR HCC OR "liver cancer" OR "liver carcinoma*" OR "hepatoma*"
I (intervention)	Keywords searched for in abstract and title	"Immune checkpoint inhibitor*" OR "immune checkpoint blockers*" OR PD-1 OR "programmed cell death 1" OR PD-L1 OR "programmed cell death ligand 1" OR CTLA-4 OR "cytotoxic T-lymphocyte-associated protein 4" OR nivolumab OR pembrolizumab OR atezolizumab OR avelumab OR camrelizumab OR durvalumab OR ipilimumab OR tislelizumab OR toripalimab OR penpulimab OR tremelimumab OR cemipilmab
C (comparison)	Keywords searched for in abstract, title	("alpha-Fetoproteins" OR alpha-Fetoprotein* OR "alpha Fetoprotein*" OR AFP OR "alpha fetal protein" OR alpha-fetal-protein)
O (outcome)	None	
S (study design)	None	
Additional limits	Limits to English language only	

Supplementary Retrieval Methods

Pubmed Search Method:

("Carcinoma, Hepatocellular" OR "hepatocellular carcinoma*" OR "hepatocellular cancer" OR "hepatic carcinoma*" OR HCC OR "liver cancer" OR "liver carcinoma*" OR "hepatoma*") AND ("Immune checkpoint inhibitor*" OR "immune checkpoint blockers*" OR PD-1 OR "programmed cell death 1" OR PD-L1 OR "programmed cell death ligand 1" OR CTLA-4 OR "cytotoxic T-lymphocyte-associated protein 4" OR nivolumab OR pembrolizumab OR atezolizumab OR avelumab OR camrelizumab OR durvalumab OR ipilimumab OR tislelizumab OR toripalimab OR penpulimab OR tremelimumab OR cemipilmab) AND ("alpha-Fetoproteins" OR alpha-Fetoprotein* OR "alpha Fetoprotein*" OR AFP OR "alpha fetal protein" OR alpha-fetal-protein)

Result: 372

Embase Search Method:

('carcinoma, hepatocellular'/exp OR 'carcinoma, hepatocellular' OR 'hepatocellular carcinoma*' OR 'hepatocellular cancer'/exp OR 'hepatocellular cancer' OR 'hepatic

carcinoma*' OR hcc OR 'liver cancer'/exp OR 'liver cancer' OR 'liver carcinoma*' OR 'hepatoma*') AND ('immune checkpoint inhibitor*' OR 'immune checkpoint blockers*' OR 'pd 1'/exp OR 'pd 1' OR 'programmed cell death 1'/exp OR 'programmed cell death 1' OR 'pd l1' OR 'programmed cell death ligand 1'/exp OR 'programmed cell death ligand 1' OR 'ctla 4'/exp OR 'ctla 4' OR 'cytotoxic t-lymphocyte-associated protein 4' OR 'nivolumab'/exp OR nivolumab OR 'pembrolizumab'/exp OR pembrolizumab OR 'atezolizumab'/exp OR atezolizumab OR 'avelumab'/exp OR avelumab OR 'camrelizumab'/exp OR camrelizumab OR 'durvalumab'/exp OR durvalumab OR 'ipilimumab'/exp OR ipilimumab OR 'tislelizumab'/exp OR tislelizumab OR 'toripalimab'/exp OR toripalimab OR 'penpulimab'/exp OR penpulimab OR 'tremelimumab'/exp OR tremelimumab OR cemipilmab) AND ('alpha-fetoproteins'/exp OR 'alpha-fetoproteins' OR 'alpha fetoprotein*' OR afp OR 'alpha fetal protein')
Result: 1497

Web of Science Search Method:
TS=("hepatocellular carcinoma*" OR "hepatocellular cancer" OR "hepatocellular cancer" OR "hepatic carcinoma*" OR HCC OR "liver cancer" OR "liver carcinoma*" OR "hepatoma*") AND TS=("immune checkpoint inhibitor*" OR "immune checkpoint blockers*" OR PD-1 OR "programmed cell death 1" OR PD-L1 OR "programmed cell death ligand 1" OR CTLA-4 OR "cytotoxic T-lymphocyte-associated protein 4" OR nivolumab OR pembrolizumab OR atezolizumab OR avelumab OR camrelizumab OR durvalumab OR ipilimumab OR tislelizumab OR toripalimab OR penpulimab OR tremelimumab OR cemipilmab) AND TS=("alpha-Fetoproteins" OR alpha-Fetoprotein* OR "alpha Fetoprotein*" OR AFP OR "alpha fetal protein" OR alpha-fetal-protein)
Result: 584

Cochrane library database Search Method:
("Carcinoma, Hepatocellular" OR "hepatocellular carcinoma*" OR "hepatocellular cancer" OR "hepatic carcinoma*" OR HCC OR "liver cancer" OR "liver carcinoma*" OR "hepatoma*") AND ("Immune checkpoint inhibitor*" OR "immune checkpoint blockers*" OR PD-1 OR "programmed cell death 1" OR PD-L1 OR "programmed cell death ligand 1" OR CTLA-4 OR "cytotoxic T-lymphocyte-associated protein 4" OR nivolumab OR pembrolizumab OR atezolizumab OR avelumab OR camrelizumab OR durvalumab OR ipilimumab OR tislelizumab OR toripalimab OR penpulimab OR tremelimumab OR cemipilmab) AND ("alpha-Fetoproteins" OR alpha-Fetoprotein* OR "alpha Fetoprotein*" OR AFP OR "alpha fetal protein" OR alpha-fetal-protein)
Result: 75
